# Catheter-based endovascular celiac and hepatic denervation for type 2 diabetes: a multicenter, open-label, single-arm study

**DOI:** 10.1038/s41392-025-02459-6

**Published:** 2025-11-13

**Authors:** Zhi Wang, Tao Pan, Weifu Lv, Jun Tang, Yan Chen, Xiangyun Zhu, Qi Zhang, Fei Jing, Han Yin, Dong Lu, Lei Zhang, Dechen Liu, Jiajun Zhao, Ling Li, Jianping Weng, Gao-Jun Teng

**Affiliations:** 1 Center of Interventional Radiology & Vascular Surgery, Department of Radiology, Zhongda Hospital, School of Medicine, Southeast University, Nanjing, Jiangsu China; 2 Department of Radiology, The First Affiliated Hospital of USTC, Division of Life Sciences and Medicine, University of Science and Technology of China, Hefei, Anhui China; 3 Department of Interventional Radiology, Shandong Provincial Hospital Affiliated to Shandong First Medical University, Jinan, Shandong China; 4 Department of Endocrinology, Institute of Endocrine and Metabolic Diseases, The First Affiliated Hospital of USTC, Division of Life Sciences and Medicine, Clinical Research Hospital of Chinese Academy of Sciences (Hefei), University of Science and Technology of China, Hefei, Anhui China; 5 Department of Endocrinology, Zhongda Hospital, School of Medicine, Southeast University, Nanjing, Jiangsu China; 6 Department of Endocrinology, Shandong Provincial Hospital Affiliated to Shandong First Medical University, Jinan, Shandong China

**Keywords:** Endocrine system and metabolic diseases, Endocrine system and metabolic diseases

## Abstract

Sympathetic hyperactivity is crucial driving factor for hyperglycemia in type 2 diabetes (T2D). We conducted a multicenter, open-label, single-arm trial to evaluate the safety and efficacy of endovascular denervation (EDN) targeting the celiac and hepatic arteries in patients with poorly controlled T2D, defined as glycated hemoglobin (HbA1c) > 7.5% and <10.5% despite treatment with metformin and at least one other antidiabetic agent (registered with the National Medical Products Administration of China and ClinicalTrials.gov, NCT05631561). A total of 37 patients were enrolled across three centers, and 30 patients underwent EDN between December 2022 and October 2023. Follow-up visits were conducted at 1, 3, 6, and 12 months. The primary endpoints were device- and/or procedure-related major adverse events (MAEs) within 30 days post-procedure and changes in HbA1c at 6 months. In treated patients, the baseline mean HbA1c was 9.0%, and the baseline mean fasting plasma glucose was 8.6 mmol/L. No MAEs were observed within 30 days. HbA1c decreased by 1.0% at 6 months (*P* < 0.001), with reductions of 1.0%, 1.2%, and 0.9% at 1, 3, and 12 months post-EDN, respectively. Time in range (3.9–10.0 mmol/L) increased by 15.4%, 14.9%, 7.6% and 11.7% at each follow-up time point. Blood pressure also showed reductions during follow-up. Adverse events were reported in 11 (37%) patients, with mild to moderate gastrointestinal symptoms being the most common. These exploratory findings provide a rationale for further evaluating the efficacy of EDN in improving glycemic control in T2D patients through randomized controlled trials.

## Introduction

The global prevalence of type 2 diabetes (T2D) is rising, with 529 million people affected in 2021 and predicted to exceed 1.31 billion by 2050.^1^ This increase underscores the growing public health burden of T2D, which contributes substantially to morbidity, mortality, and healthcare costs worldwide. Over the past decades, numerous pharmacological therapies have been developed and optimized to manage hyperglycemia, including recent advancements such as glucagon-like peptide-1 (GLP-1) receptor agonists.^2^ Despite the availability of numerous safe and effective pharmacological therapies, a significant proportion of patients experience poor glycemic control.^3^ Much failure of the pharmacological strategy to attain adequate glycemic control is attributed to both physician inertia and patient non-compliance and non-adherence to the pharmacological therapy.^4^ In addition, the progressive nature of T2D often necessitates multiple medications,^5^ which further complicates adherence and reduces long-term treatment effectiveness. These challenges underscore the limitations of current strategies and the urgent need to develop alternative approaches that can provide durable glycemic control independent of daily patient compliance.

Sympathetic hyperactivity is a representative pathophysiological change of T2D, promoting hepatic gluconeogenesis and inhibiting insulin secretion, both of which contribute to hyperglycemia.^6^ Beyond its metabolic role, sympathetic overactivity also aggravates cardiovascular, renal, and inflammatory complications, making it a unifying target for intervention under the context of T2D.^7^ Therapeutics targeting the sympathetic nervous system (SNS) have been studied across various diseases. Renal denervation (RDN), extensively researched and evidence-backed, has shown efficacy in lowering sympathetic activity by disrupting the renal sympathetic nerves.^8^ Owing to the involvement of sympathetic hyperactivity, RDN has been explored as a potential intervention for managing T2D,^9,10^ in addition to its application in resistant hypertension.^11,12^ However, although a preclinical research suggested that RDN improve systemic glucose metabolism,^10^ clinical evidence did not support its effectiveness in glycemic control.^13^ This gap indicates that renal sympathetic nerves alone may not be the most relevant target for metabolic modulation, and sympathetic pathways directly regulating hepatic glucose output and insulin secretion should be prioritized.

According to the hyperglycemic effect of SNS on the liver and islets, our team has focused on targeting the sympathetic innervation of these organs by shifting the ablation target from the renal artery to the celiac artery and hepatic arteries, where sympathetic nerves to the liver and islets are primarily distributed.^14,15^ This novel approach, termed endovascular denervation (EDN), is designed to disrupt sympathetic input to metabolic organs most critical for glucose homeostasis. Our preliminary first-in-human studies of EDN have shown promising outcomes for glycemic control in patients with T2D, demonstrating improvements in glycated hemoglobin (HbA1c) and liver function.^16^ Notably, liver function improvement may also indicate potential benefits in patients with concomitant metabolic dysfunction–associated steatotic liver disease (MASLD), a highly prevalent comorbidity in T2D. These findings shed a ray of light on neuromodulation as an interventional strategy for glycemic control. However, given the small sample size (*n* = 11) and limited diversity of clinical indices, further evidence from larger studies is necessary to validate safety and efficacy.

This study aimed to assess the safety and effectiveness of therapeutic celiac and hepatic EDN in patients with T2D who have poor glycemic control on standard antidiabetic therapies. By expanding sample size, incorporating a broader range of metabolic endpoints, and systematically evaluating adverse events, this trial aims to strengthen the evidence base for EDN. The study is among the first to evaluate targeted denervation of the splanchnic sympathetic nerves in humans with T2D, providing new insights into neuromodulation as a complementary treatment strategy, offering a durable, compliance-independent intervention that addresses the neurogenic drivers of hyperglycemia.

## Results

### Baseline characteristics

**Fig. 1:**
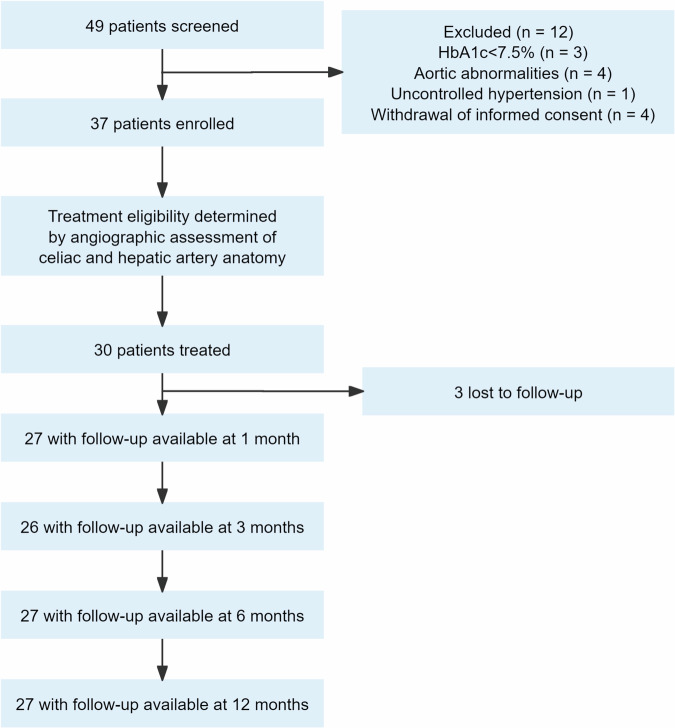
Trial profile. HbA_1c_ = glycated hemoglobin

Forty-nine patients were screened, among which 12 patients were excluded from the procedure according to the inclusion/exclusion criteria. Thirty-seven patients were enrolled in preparation for celiac and hepatic EDN, and 30 of them finally underwent celiac and hepatic EDN (Fig. 1).

The treated patients had a mean age of 51 years (SD, 7.0; range, 32–61 years), and 27% (*n* = 8) were female (Table 1). Concomitant illnesses, including hypertension, hyperlipidemia, and metabolic dysfunction-associated steatotic liver disease (MASLD), were listed (Table 1). They had a mean baseline alanine transaminase of 35.3 U/L (SD, 22.6).

**Table 1:** Baseline characteristics of patients

Characteristics	All patients (*N* = 37)	Patients undergoing procedure (*N* = 30)	Patients not undergoing procedure (*N* = 7)
Age, years			
Mean (SD)	51 (7)	51 (7)	52 (6)
Range	32–61	32–61	43–60
Sex			
Female	10 (27%)	7 (23%)	3 (43%)
Male	27 (73%)	23 (77%)	4 (57%)
HbA_1c_, %			
Mean (SD)	9.0 (0.8)	9.0 (0.8)	9.2 (1.0)
Range	7.8–10.5	7.8–10.5	8.0–10.5
Body weight, kg			
Mean (SD)	74.5 (11.2)	75.8 (10.2)	69.0 (14.6)
Range	48.0–95.0	58.0–95.0	48.0–83.0
BMI, kg/m^2^			
Mean (SD)	26.7 (3.5)	27.0 (3.6)	25.4 (3.2)
Range	20.5–39.5	21.3–39.5	20.5–29.1
Duration of diabetes, years			
Mean (SD)	7 (3)	7 (3)	7 (3)
Range	2–13	2–13	2–11
FPG, mmol/L			
Mean (SD)	8.5 (2.0)	8.5 (1.9)	8.3 (1.4)
Range	5.2–15.1	5.2–15.1	6.3–10.8
SBP, mmHg			
Mean (SD)	129 (12)	130 (12)	129 (14)
Range	108–157	108–157	113–146
DBP, mmHg			
Mean (SD)	84 (11)	84 (10)	84 (13)
Range	66–99	66–99	66–99
Concomitant disease			
Hypertension	12 (32%)	11 (33%)	1 (14%)
Hyperlipidemia	21 (57%)	18 (60%)	3 (43%)
MASLD	10 (27%)	10 (33%)	0 (0%)
Oral antidiabetic drugs no.	2 (2–3)	2 (2–3)	2 (2–3)
Metformin	37 (100%)	30 (100%)	7 (100%)
Sulfonylureas	31 (84%)	27 (87%)	4 (57%)
Thiazolidinediones	3 (8%)	1 (3%)	2 (29%)
SGLT2 inhibitor	3 (8%)	3 (10%)	0 (0%)
α-glucosidase inhibitor	2 (5%)	1 (3%)	1 (14%)
Insulin	12 (32%)	9 (30%)	3 (43%)
Other concomitant medications			
Angiotensin-converting enzyme inhibitors	2 (5%)	2 (6.7%)	0 (0%)
Beta blocker	1 (3%)	1 (3%)	0 (0%)
Calcium channel blocker	6 (16%)	5 (17%)	1 (14%)
Diuretics	2 (5%)	2 (7%)	0 (0%)
Aspirin	3 (8%)	2 (7%)	1 (14%)
Statin	1 (3%)	0	1 (14%)

Data are mean (SD) or number (%)

HbA1c at enrollment was 9.0% (SD, 0.8), with a fasting plasma glucose of 8.5 mmol/L (SD, 1.9). Antidiabetic drugs included metformin, thiazolidinediones, sulfonylureas, SGLT2 inhibitors, α-glucosidase inhibitor, and insulin (Table 1). The baseline profile demonstrated that the enrolled patients represented a typical population with poorly controlled T2D and multiple metabolic comorbidities.

### Safety outcomes

Safety outcomes were analyzed to evaluate peri-procedural safety and adverse events associated with EDN. Among the 30 treated patients, the median procedure time from initiation to completion of radiofrequency delivery was 27 min (IQR, 17–38 min). On average, a total of 24.6 (SD, 5.1) ablations were conducted, including 16.8 (SD, 5.7) in the hepatic artery and 7.8 (SD, 3.7) in the celiac artery. Denervation was accompanied by moderate abdominal pain in 10 patients. The pain was relieved in 9 patients after administration of analgesics and sedative drugs (hydromorphone hydrochloride 2 mg in 9 patients and promethazine 25 mg in 1 patient), except for one patient who required paracetamol-dihydrocodeine after the procedure.

Among the treated patients, one patient missed the 1-month follow-up visit due to scheduling issues but completed all subsequent visits. Three patients were lost to follow-up at all time points, with one who could not be contacted despite repeated attempts and two who declined further participation for personal reasons. One of the primary endpoints was the composite of MAE related to the study device and/or the procedure within 30 days, which was not observed. Adverse events were reported by 11 (37%) patients that underwent EDN (Table 2). The most common category of adverse events was gastrointestinal adverse events, which occurred in 9 (30%) of 30 participants after procedure. All were mild or moderate in severity, with no associated abnormalities in laboratory tests. Importantly, none of the cases exhibited clinical features suggestive of serious abdominal pathology or required further diagnostic evaluation. Symptoms in five patients resolved within one week, with or without treatment. There are no clinically significant hypoglycemic episodes reported. One case of mild skin allergy was recorded and was evaluated as associated with the procedure and resolved spontaneously with no treatment. One adverse event of arterial spasm was recorded during the procedure, and resolved without subsequent complications. At the 6-month follow-up, CT angiography showed no evidence of arterial stenosis or other abnormalities. These results demonstrate that EDN can be performed safely and feasibly, with no major adverse events or procedure-related mortality observed.

**Table 2:** Summary of adverse events

Adverse events	No. of patients (%)
Gastrointestinal adverse events	9 (30%)
Nausea	2 (7%)
Constipation	1 (3%)
Diarrhea	1 (3%)
Vomiting	2 (7%)
Abdominal pain	3 (10%)
Dyspepsia	2 (7%)
Arterial spasm	1 (3%)
Skin allergy	1 (3%)

### Efficacy outcomes

**Fig. 2:**
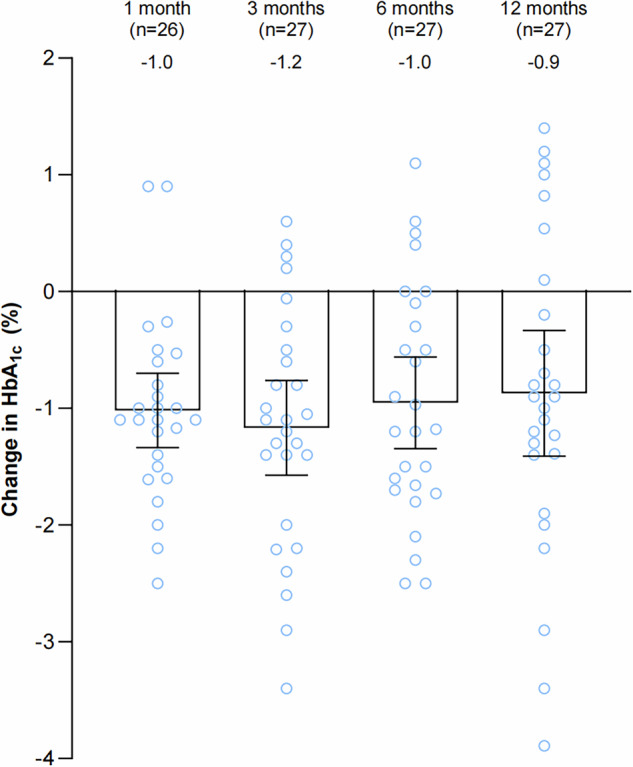
Change in HbA_1c_ (95% CI) at 1, 3, 6, and 12 months. Numbers in parentheses indicate patients who had attended each predefined visit

To determine the efficacy of EDN in patients with T2D, we assessed HbA1c at 1, 3, 6, and 12 months (Fig. 2). As the primary endpoint, HbA1c levels showed a significant mean reduction of −1.0% (−1.3 to −0.6) at 6 months compared to baseline. The significant reduction in HbA1c observed 1 month after the procedure persisted through subsequent assessments up to 12 months, with reductions of –1.0% (95% CI, −1.3 to −0.7), −1.2% (95% CI, −1.6 to −0.8), −1.0% (95% CI, −1.3 to −0.6), and −0.9% (95% CI, −1.4 to −0.3) at 1, 3, 6, and 12 months, respectively. Repeated measures with pair-wise comparison showed that HbA1c levels were significantly lower at each follow-up time point than those before procedure (*P* < 0.001, *P* < 0.001, *P* < 0.001, *P* = 0.010). Nineteen of 27 patients (70%) who were followed up had HbA1c reductions of at least 0.3% (response) at 6 months, which is a noninferiority criterion and represents a clinically meaningful reduction, as established by the U.S. Food and Drug Administration^17^ and the European Medicines Agency^18^ for evaluating the efficacy of new diabetes medications. Although not addressed in the protocol, we obtained and analyzed the 1-year HbA1c data for patients who did not undergo the procedure. After excluding two patients who withdrew informed consent, the remaining five patients showed a mean change in 1-year HbA1c of 0.1% (95% CI, −0.4 to 0.5).

In treated patients, univariate analysis failed to show a clear association between HbA1c reduction of 0.3% or more and any of the following: age, sex, history of hypertension, dyslipidemia, MASLD, baseline blood pressure, baseline liver function, baseline heart rate, baseline number of antidiabetic medications, baseline antidiabetic drug types, or number of ablations.

**Fig. 3:**
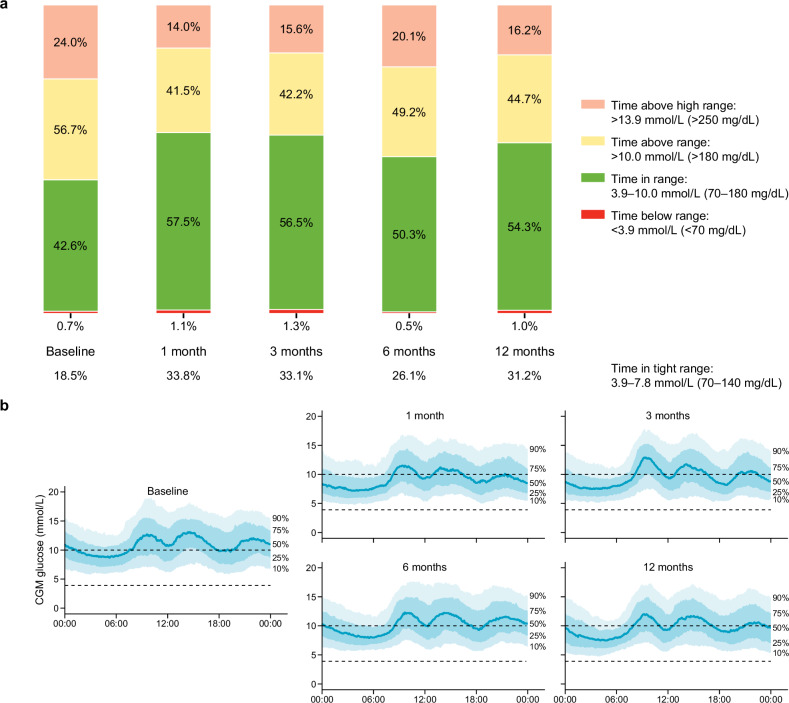
CGM observations. **a** Time below range, time in range, time above range, time above high range, and time in tight range (percentage of readings); note that time above high range is a subcategory of time above range. Baseline: *n* = 30; 1 and 3 months: *n* = 26; 6 and 12 months: *n* = 27. **b** 24-h CGM profiles. The dark blue line signifies the median, the blue bands represent the 10–90th and 25–75th centiles, and the dashed lines represent the blood glucose target range of 3.9–10.0 mmol/L

All patients had 14-day CGM at baseline and at follow-ups after celiac and hepatic EDN. Fourteen-day mean glucose and overnight glucose were significantly decreased at all time points (Supplementary Table 2). Mean TIR changes were 15.4% (95% CI, 7.0 to 23.8), 14.9% (95% CI, 6.1 to 23.6), 7.8% (95% CI, 0.3 to 14.9), and 11.7% (95% CI, 4.2 to 19.2) at 1, 3, 6, and 12 months, respectively. Mean TAR changes were −15.8 (95% CI, −24.7 to −6.9), −15.6 (95% CI, −24.6 to −6.5), −15.2 (95% CI, −15.2 to −0.1), −12.1 (95% CI, −20.0 to −4.1) (Fig. 3). In HbA1c responders (HbA1c reduction > 0.3%, *n* = 19), mean 14-day TIR changes were 15.6% (95% CI, 5.0 to 26.1), 15.0 (95% CI, 6.6 to 23.4), 9.3% (95% CI, 0.9 to 17.6), and 17.5% (95% CI, 9.5 to 25.4). HbA1c changes at the corresponding time points were −1.3 (95% CI, −1.6 to −1.0), −1.5 (95% CI, −2.0 to −1.1), −1.5 (95% CI, −1.8 to −1.2), and −1.4 (95% CI, −1.9 to −0.8) in these patients. In HbA1c non-responders (HbA1c reduction < 0.3%, *n* = 8), mean TIR changes were 15.0% (95% CI, −2.6 to 32.6), 12.5% (95% CI, −17.7 to 42.7), 4.1% (95% CI, 13.2 to 21.3), and −1.9% (95% CI, −16.9 to 12.9). Mean HbA1c changes at the corresponding time points were −0.5% (95% CI, −1.2 to 0.3), −0.3% (95% CI, −0.9 to 0.3), 0.3% (95% CI, −0.1 to 0.7), and 0.3% (95% CI, −0.5 to 1.1) in these patients. Furthermore, EDN led to significant improvements in time in tight range (TITR, 3.9–7.8 mmol/L) at all time points, as well as in the standard deviation of glucose and mean amplitude of glycemic excursions (MAGE) at 1 month. However, CGM-based glucose variability indices—specifically, the standard deviation and coefficient of variation—did not demonstrate significant improvement over the course of follow-up (Supplementary Table 1). Fasting plasma glucose showed a transient increase at 6 months. In addition, OGTT-derived glucose levels showed no significant changes from baseline at any time point (Table 3 and Supplementary Fig. 1). Numerical increases in triglycerides, total cholesterol, and low-density lipoprotein cholesterol, although not significant, were observed after the procedure, while high-density lipoprotein cholesterol showed a significant increase (Table 3).

**Table 3:** Comparison of baseline and follow-up characteristics

Characteristics		Baseline (*n* = 30)	1 month (*n* = 26)	3 month (*n* = 27)	6 months (*n* = 27)	12 months (*n* = 27)	*P*
Glycemic indexes	HbA1c—%	9.0 (0.8)	8.0 (0.7)	7.8 (1.0)	8.0 (1.0)	8.1 (1.0)	<0.001
	FPG—mmol/L	8.5 (1.9)	8.6 (2.1)	9.1 (2.0)	10.6 (2.1)	9.1 (2.5)	0.001
Anthropometric and physiological measurements	SBP—mmHg	129.8 (11.8)	123.8 (12.2)	125.0 (13.5)	124.4 (11.7)	124.1 (11.3)	0.070
	DBP—mmHg	84.4 (9.9)	81.5 (8.0)	81.3 (8.4)	78.9 (8.6)	79.2 (8.4)	0.006
	Heart rate	78.4 (8.8)	78.7 (8.6)	78.6 (9.8)	83.7 (9.4)	78.8 (9.9)	0.020
	Weight—kg	75.8 (10.2)	75.0 (10.2)	75.7 (9.3)	76.6 (9.1)	75.4 (9.2)	0.060
	BMI—kg/m^2^	27.0 (3.9)	26.7 (3.7)	27.0 (3.5)	27.3 (3.2)	26.8 (3.4)	0.053
Liver function	ALT—U/L	35.3 (22.6)	29.5 (14.9)	33.6 (22.2)	36.1 (39.4)	29.9 (15.2)	0.301
	AST—U/L	27.3 (13.3)	23.5 (9.7)	27.0 (17.8)	32.1 (45.8)	24.1 (8.6)	0.117
	GGT—U/L	38.9 (24.7)	36.9 (21.0)	41.2 (23.4)	43.7 (31.3)	33.2 (13.5)	0.085
	ALP—U/L	86.0 (27.8)	77.4 (26.2)	79.7 (25.7)	78.6 (24.8)	79.9 (26.1)	0.109
Blood lipid	TG—mmol/L	2.3 (2.2)	2.7 (2.5)	2.7 (2.4)	2.4 (3.0)	2.0 (1.6)	0.430
	TC—mmol/L	4.6 (0.9)	4.5 (0.7)	4.8 (0.8)	4.8 (0.8)	4.8 (0.9)	0.164
	LDL— mmol/L	2.8 (1.0)	2.6 (0.8)	2.9 (0.8)	2.9 (0.9)	2.9 (0.8)	0.111
	HDL—mmol/L	1.1 (0.3)	1.1 (0.3)	1.2 (0.3)	1.2 (0.3)	1.1 (0.3)	0.005
Catecholamine	E—pg/mL	38.8 (25.1)	43.0 (30.4)	37.0 (21.0)	47.5 (27.4)	38.0 (26.2)	0.229
	NE—pg/mL	160.9 (88.3)	370.7 (389.5)	263.0 (157.0)	330.0 (256.0)	200.8 (200.2)	0.131
Heart rate variability	Low frequency—ms^2^	339.5 (343.9)	303.7 (224.4)	NA	240.9 (173.6)	NA	0.243
	High frequency—ms^2^	182.0 (154.5)	147.6 (94.7)	NA	138.0 (97.7)	NA	0.203
	LF/HF	2.2 (1.4)	2.3 (1.5)	NA	2.1 (1.5)	NA	0.670

*ALP* alkaline phosphatase, *ALT* alanine aminotransferase, *AST* aspartate aminotransferase, *AUC* area under the curve, *BMI* body mass index, DBP diastolic blood pressure, *E* epinephrine, *FPG* fasting plasma glucose, *GGT* gamma-glutamyl transferase, *HbA*_1c_ glycated hemoglobin, *HDL* high-density lipoprotein, *HF* high frequency, *LDL* low-density lipoprotein, *LF* low frequency, *NE* norepinephrine, *SBP* systolic blood pressure, *TAR* time above range, *TBR* time below range, *TC* total cholesterol, *TG* triglycerides, *TIR* time in range, *Tbil* total bilirubin

At baseline, patients were administered a median of 2 (IQR, 2-3) medications, and this number remained unchanged during follow-up. Patients and physicians were instructed not to change medications, except if clinically necessary. Nonetheless, 10 of 27 patients changed their medications during follow-up, including four patients in whom medications were increased and six in whom medications were reduced (Table 4). Among patients whose medications were increased, three had a reduction in HbA1c greater than 0.3% before medication change. Among the six patients who reduced their medication, three had achieved optimal HbA1c control (<7.0%) and two had HbA1c levels at 7.0–7.5% before medication reduction. One patient independently reduced the insulin dosage after a 2.2% HbA1c reduction was observed at the 1-month follow-up (from 10.7% to 8.5%). The effectiveness of the procedure to reduce HbA1c was preserved even after censoring data from patients who increased the antidiabetic medications: mean HbA1c reductions were −1.1 (95% CI, −1.4 to −0.8), −1.2 (95% CI, −1.6 to −0.8), −1.0 (95% CI, −1.4 to −0.5), and −1.1 (95% CI, −1.7 to −0.5) at 1, 3, 6, and 12 months, respectively.

**Table 4:** Summary of medication changes

Patients	Direction of medication change	Medications changed	Daily dose change	HbA1c (%)		
				Baseline	Last value before medication change	6 months
1	Decrease	Insulin	from 15 to 4 IU	9.1	6.2	6.6
2		Insulin	from 20 to 10 IU	10.0	7.4	7.5
3		Glimepiride	from 4 to 0 mg	8.5	6.8	7.0
4		Metformin	from 1500 to 1000 mg	10.7	8.5	8.4
		Glimepiride	from 4 to 0 mg			
		Insulin	from 30 to 13 IU			
5		Metformin	from 1000 to 500 mg	9.6	6.9	7.8
		Gliclazide	from 120 to 60 mg			
6		Glimepiride	from 4 to 2 mg	8.4	7.3	6.8
7	Increase	Insulin	from 14 to 18 IU	7.9	7.6	6.7
8		Insulin	from 14 to 18 IU	9.2	7.6	7.5
9		Insulin	from 24 to 30 IU	8.3	8.5	8.8
10		Metformin	from 1000 to 1500 mg	9.3	8.5	8.7

We assessed the systemic SNS activity by measuring HRV at baseline, 1 month, and 6 months, and plasma norepinephrine at all follow-up time points. Low frequency, high frequency, and the ratio of low frequency and high frequency remained unchanged from baseline at predetermined time points after the procedure. Heart rates were 79 bpm at baseline and 79, 79, 84, and 79 bpm at 1, 3, 6, and 12 months, respectively (Table 3). Norepinephrine did not show significant change throughout the follow-up period. However, celiac and hepatic EDN was associated with a trend toward systolic blood pressure reduction and a statistically significant decrease in diastolic blood pressure. Specifically, the mean changes from baseline to each time points were −5.7 (95% CI, −11.3 to −0.1), −4.2 (95% CI, −10.2 to 1.7), −4.8 (95% CI, −11.2 to 1.6), and −5.1 (95% CI, −11.4 to 1.2) mmHg for systolic blood pressure and −3.1 (95% CI, −6.9 to 0.7), −3.0 (95% CI, −7.8 to 1.7), −5.4 (95% CI, −10.0 to −0.8), and −5.1 (95% CI, −10.3 to 0.1) mmHg for diastolic blood pressure.

Baseline and follow-up liver function data were available at each time point. In 7 (23.3%) patients with elevated baseline ALT ( > 40 U/L), 4 had reductions to normal range at 6 months. This reduction occurred together with a substantial drop (>1.5%) in HbA1c in three patients. Those with abnormal baseline ALT levels (>40 U/L) tend to have a greater decrease in ALT (−20.0 ± 28.4 vs. −3.2 ± 10.2, *P* = 0.029). Taken together, EDN significantly improved glycemic control and CGM-derived glucose metrics, with potential favorable effects on diastolic blood pressure and liver function.

## Discussion

We present clinical findings of celiac and hepatic EDN using a catheter-based device for the treatment of patients with T2D. In this trial, we showed a good safety profile of this therapy. No severe and long-term adverse events resulted from the procedure. Celiac and hepatic EDN resulted in a clinically relevant reduction in HbA1c that was evident as early as 1 month, was further reduced at 3 months, and persisted through subsequent assessments. Furthermore, celiac and hepatic EDN resulted in reduced blood pressure.

Although HbA1c reflects average glycemic control over approximately 3 months, improvement was already observed at 1 month postprocedure, suggesting that the early postprocedural period had a meaningful impact on the 1-month HbA1c value. Consistent with this observation, HbA1c continued to decline at 3 months, likely reflecting the additional contribution of improved glycemic control during the full postprocedural 1–3 month period. Corresponding to the improvements in HbA1c, the GCM data revealed benefits of EDN in glycemic control, along with glycemic fluctuation and excursion. These observations indicated potential postprandial glucose-lowering effect. This effect underscored the mechanism of postprandial glucose disposal, such as hepatic glycogen synthesis, which was suggested by a preclinical canine study using surgical hepatic denervation.^19^ Although fasting glucose levels did not show significant improvement, we used mean overnight glucose levels (0:00–6:00 a.m.) in CGM as an alternative to reflect glucose at fasting state and observed significant decrease. OGTT data, similar to fasting glucose levels, did not show improvements after the procedure. On the one hand, fasting blood glucose and OGTT may not be as reliable as HbA1c and CGM in assessing glucose control, as studies have shown poor reproducibility of intraindividual fasting glucose and OGTT results^20^. These tests primarily reflect short-term glucose metabolism and may not capture day-to-day fluctuations or the broader patterns of chronic glucose regulation. Since glucose variability did not significantly change after the procedure, it is plausible that short-term glucose excursions persisted despite improvements in average glycemic metrics. On the other hand, white coat hyperglycemia—resulting from heightened sympathetic activity due to stress—can also influence short-term glucose metabolism^21^ and potentially skew results in OGTT and fasting blood glucose. Therefore, it is essential to interpret these results within the context of sympathetic modulation and consider the predictable alteration in the sympathetic response to stress after this procedure. Another factor that could potentially influence the results is that the median 6-month follow-up time fell at 2 days after the long holidays of the Chinese Spring Festival, known to pose challenges for glycemic control.^22^ Specifically, 14 patients had their 6-month follow-ups within three months after the Chinese New Year, a period associated with significantly higher fasting blood glucose levels.^22^ Thus, the 12-month results are likely less influenced by this factor.

The HbA1c-lowering effect of EDN was preserved in most cases among those with medication changes. This supports the potential of EDN as a complementary intervention that may enable de-intensification of pharmacologic therapy in selected patients. While the study included commonly used antidiabetic agents, GLP-1 receptor agonists were not incorporated due to limited use at the time of trial design. Preclinical studies indicate that sympathetic innervation suppresses GLP-1 secretion from intestinal L-cells^23^. Similarly, RDN has been shown to reduce renal SGLT2 expression, while SGLT2 inhibitors may lower sympathetic tone via both renal and central mechanisms^24^. These findings highlight the close interplay between the autonomic and endocrine systems in glucose regulation, and suggest that EDN may act synergistically with GLP-1 receptor agonists and SGLT2 inhibitors—an area worthy of future investigation.

Assessing organ-specific sympathetic activity remains a significant challenge, as methods such as liver biopsy and NE spillover measurement are invasive and technically demanding.^25^ Given these limitations, we instead evaluated whether EDN had an impact on systemic sympathetic tone by measuring plasma NE levels and HRV, primarily to help rule out a major systemic effect. While both measures are widely used indicators of systemic sympathetic activity, they do not provide information on regional or organ-specific sympathetic responses.^26^ We observed unaltered plasma NE levels and HRV post-EDN. While HRV offers a simplified association with the autonomic regulation of the sinoatrial node, it has limitations in accurately representing the autonomic functions of other organs.^27^ These findings offer insights into EDN’s impact on SNS, and further underscore the need for improved techniques to better quantify the degree of denervation in RDN and EDN. Potential approaches include less invasive norepinephrine spillover assessment that accounts for the liver’s dual blood supply, endovascular electrophysiological recording, advanced functional imaging techniques, and the use of phenomics to identify metabolic phenotypes associated with organ-specific sympathetic activity.

During the follow-up periods, patients experienced a slight numerical attenuation of achieved reduction in HbA1c, suggesting the counter-regulatory mechanisms, since glucose homeostasis involves complex coordination across multiple organs and systems.^28^ Another possible explanation is nerve regeneration. While direct evidence in humans is lacking, preclinical studies of RDN have shown timelines of reinnervation, including neuromatous tangle formation in swine at 90 days^29^, partial functional recovery as early as 12 weeks in rats^30^, and near-complete recovery of renal NE levels at 11 months in sheep^31^. Due to technical limitation, no definitive reinnervation has been verified in clinical RDN trials, while blood pressure–lowering effects have persisted for up to 3 years^32^. Given the distinct innervation of the liver and pancreas compared to the kidney, the potential for nerve regrowth, the long-term durability of efficacy, and the need for possible re-intervention warrant further investigation through long-term follow-up and dedicated preclinical studies.

Our previous pilot preliminary study demonstrated improved liver function post-treatment.^16^ The lack of change in the whole cohort of this study may be attributed to normal baseline liver function (mean ALT: 35.3 U/L). Nonetheless, we observed a non-significant numerical decline in all liver enzymes over time. Meanwhile, patients with higher baseline liver function tend to benefit from celiac and hepatic EDN. This observation prompts further investigation into specific changes in the liver, such as steatosis. To address this question, in an investigator-initiated parallel study (NCT05673668, currently under review), we used magnetic resonance imaging to quantify liver fat content. A significant reduction was observed at 6 months post-EDN, from 11.3% to 8.6%. Moreover, this improvement was significantly correlated with the reduction in HbA1c. This may provide further evidence to whether this procedure improved liver steatosis and insights into the underlying mechanism.

Laboratory tests indicated significant increases in HDL. HDL serves multiple functions beyond cholesterol transport, including antioxidant, anti-inflammatory, and immune-regulating activities.^33^ Although pharmacologically increasing HDL levels has not consistently demonstrated clinical benefits, research is ongoing to develop therapies that specifically enhance HDL-mediated reverse cholesterol transport.^33^ Whether the metabolic effects of EDN involve HDL regulation and whether the increase in HDL leads to clinical benefits will be addressed in future studies.

Another key finding was the reduction in blood pressure. RDN lowers blood pressure by disrupting efferent and afferent renal nerves, reducing central and renal sympathetic activity, thereby promoting sodium and water excretion, and inhibiting reflex sympathetic activation.^34^ Reasonably, the anti-hypertensive effect of celiac and hepatic EDN is distinct, likely due to its metabolic impact, as improvements of glycemic control and steatosis can contribute to lower blood pressure.^35,36^ However, single-time office blood pressure measurements have inherent limitations. Since ambulatory blood pressure monitoring provides a more accurate and comprehensive assessment of blood pressure changes, future studies incorporating this method are warranted to further evaluate the hemodynamic effects of EDN.

A key limitation of this study is the relatively small sample size and single-arm design, which means that potential placebo effects and regression to the mean cannot be excluded. Another limitation was the absence of patients using GLP-1 receptor agonists, as this medication was not widely used in patients with T2D in China at the time the trial was initiated. Additionally, we assessed sympathetic activity using plasma NE levels and HRV, which may not sufficiently reflect the local activity of the liver. Furthermore, the absence of pivotal measurements, such as insulin and hepatic steatosis, limits the ability to elucidate the mechanisms underlying the observed improvement in glycemic control. Finally, from both safety and efficacy perspectives, the currently unexplored effects of denervation of organs supplied by the celiac artery, such as gastric motility, pancreatic enzymes, and splenic immune responses, should be systematically evaluated in future investigations. An ongoing study evaluating these parameters may provide further insight into the effects of EDN in this context.

This study demonstrates that a simple, brief, catheter-based procedure to ablate celiac and hepatic sympathetic nerves can be performed safely without serious complications and could result in persistent improvements in glycemic control, HDL profile, and blood pressure in T2D. To move beyond the current hypothesis-generating stage, future studies are needed to validate these findings in randomized controlled trials with larger cohorts and long-term follow-up, and to clarify the underlying mechanisms of the intervention.

## Materials and methods

### Ethics

This single-arm trial was approved by the ethics review committees of Zhongda Hospital, Southeast University (2022ZDSYLL281-Y01, August 31, 2022), the First Affiliated Hospital of University of Science and Technology of China (2023-104, March 7, 2023), and Shandong Provincial Hospital Affiliated to Shandong First Medical University (2023-03, March 9, 2023). This study was carried out in alignment with local regulations, Good Clinical Practice guidelines, and Declaration of Helsinki. All patients provided written informed consent. This trial was registered with the National Medical Products Administration of China and ClinicalTrials.gov on November 30, 2022, with the number NCT05631561.

### Study design and participants

The centers were engaged in competitive patient recruitment, and participants were enrolled based on eligibility criteria. Patients were treated with celiac and hepatic EDN between December 27, 2022, and October 18, 2023. Written informed consent was obtained from all patients. The inclusion and exclusion were applied using a combination of standardized screening methods, including laboratory tests, medical imaging, and detailed medical history review. In brief, patients were eligible if they were >18 and <65 years old; were diagnosed with T2D for 1–15 years; had an glycated hemoglobin (HbA1c) of >7.5% and <10.5%, despite being treated with metformin (daily dose ≥1000 mg) with 1–3 oral antidiabetic drugs (including sulfonylureas/glinides, thiazolidinediones, α-glucosidase inhibitors) and/or insulin for more than 3 months; had body mass index of ≥18 and ≤40 kg/m^2^; understood the requirements and treatments of the trial, agreed to and were able to complete all follow-up assessments required for the trial, and signed informed consent before any special trial-related tests and treatments were performed. Patients with type 1 diabetes, secondary diabetes, severe complications of T2D, severe dysfunction of the heart, liver, kidneys, or pancreas, autonomic neuropathy, severe systemic infections, autoimmune diseases, acute illness phases, malignancies, hematological disorders, or any condition deemed unsuitable for this procedure by the physician were excluded from the trial. Details of inclusion and exclusion criteria are listed in supplemental information protocol synopsis.

### Intervention and outcome measurement

The procedural details of celiac and hepatic artery EDN are presented in the supplemental information. Briefly, after confirming eligibility under angiography, the treatment catheter (Netrod, Brattea, China) was introduced into the celiac and hepatic arteries using an 8-Fr sheath with Seldinger technique via femoral access. We applied 60 °C, discrete, radiofrequency ablations lasting up to 120 s each to obtain at least 18 ablations, with up to 6 electrodes delivering energy simultaneously during each session. During ablation, the catheter system monitored the temperature and impedance, altering radiofrequency energy delivery in response to a predetermined algorithm. To achieve broader coverage of pancreatic sympathetic innervation, the celiac artery was ablated in addition to the hepatic artery, as this could compensate for insufficient denervation of the pancreatic tail that may occur with hepatic artery ablation alone^19^. All the patients underwent angiogram right after the procedure to evaluate potential vascular complications. The procedures were performed at each center by interventional radiologists with more than 30 years of experience. Patients then underwent subsequent follow-ups at 1, 3, 6, and 12 months.

Primary outcomes included (1) device- and/or procedure-related major adverse events (MAE) within 30 days, which were assessed descriptively to evaluate procedural safety without formal hypothesis testing, and (2) the change in HbA1c at 6 months post-procedure compared to baseline. MAEs were defined as follows: severe hypoglycemia or hyperglycemia requiring hospitalization; vascular complications requiring surgical repair, endovascular intervention, thrombin injection, or blood transfusion; celiac/hepatic artery dissection or perforation requiring intervention; clinically significant embolic events resulting in end-organ damage or requiring intervention to prevent end-organ damage; acute liver injury or liver failure; and all-cause mortality. A 30-day time frame was selected, rather than a 12-month window, to focus on capturing MAEs most likely attributable to the procedure. Secondary endpoints included changes from baseline in HbA1c at 1, 3, and 12 months, fasting plasma glucose, time in range (3.9–10.0 mmol/L) from continuous glucose monitoring, blood pressure, and other safety profiles. Treated patients were analyzed per protocol, while the 12-month HbA1c levels of untreated patients were assessed and presented post hoc. For safety analysis, all adverse events were recorded by clinical research coordinators and reviewed by the trial principal investigators.

Data collection and laboratory tests were conducted independently at each center according to standard clinical protocols. Baseline measurements consisted of vital signs, physical examination, review of medications, laboratory tests, and pregnancy test, as appropriate. Assessments at each follow-up time point consisted of vital signs, physical examination, review of medications, laboratory tests, continuous glucose monitoring (CGM), and surveillance for adverse events. Patients were instructed not to change their medications unless medically necessary, as determined by their physicians. Investigators asked patients about their medication adherence at each follow-up time point, and any instances of non-compliance were documented.

CGM was performed over a 14-day period using a glucose-monitoring device (SIBIONICS, China) at each follow-up time point, with measurements taken every 5 min. The parameters assessed included mean glucose, mean overnight glucose (0:00–6:00 a.m.), TIR (3.9–10.0 mmol/L), time in tight range (3.9–7.8 mmol/L), time above range (>10.0 mmol/L), time above high range (>13.9 mmol/L), time below range (<3.9 mmol/L), coefficient of variation of glucose levels, standard deviation of glucose levels, largest amplitude of glycemic excursions (LAGE), mean amplitude of glycemic excursions (MAGE), and mean of daily differences (MODD). Oral glucose tolerance test (OGTT) was conducted before the procedure, and at 6 and 12 months after it. Plasma glucose was measured before and at 30, 60, 90, and 120 min after the intake of 300 ml of 75-g oral glucose solution. The area under curve of plasma glucose was calculated using the incremental method. Heart rate variability (HRV) was measured over 24 h at baseline, and at 1 and 6 months after the procedure using a 24-h Holter device (TES010, THOTH, China). Low frequency, high frequency, and the ratio of low frequency and high frequency were measured. CT angiography was performed at baseline and 6-month follow-up after the procedure. Patients with arterial abnormalities at 6 months were further evaluated using digital subtraction angiography for confirmation.

### Statistical analysis

The sample size was determined based on the hypothesis that patients would experience a reduction in HbA1c at 6 months. To achieve a predefined 1.0% reduction in HbA1c, with a standard deviation of 1.5%, assuming a two-sided alpha level of 0.05 and a power of 90%, a sample size of 26 would be required to yield a lower two-sided 95% confidence bound of approximately 0. Considering a 10% dropout rate, the estimated sample size is 29. Ultimately, a total of 30 patients were treated. All treated patients were analyzed for safety profile, and those with follow-up data were analyzed for efficacy. To assess efficacy, we analyzed changes in HbA1c from baseline to 6 months, with statistical tests using repeated measures analysis of variance (ANOVA), followed by pairwise comparisons with Bonferroni correction. We calculated mean changes in HbA1c and TIR (3.9–10 mmol/L), with 95% CIs from baseline at each follow-up time point. We performed analysis ignoring changes of medications and then did another analysis with censored data at times of any increase in medication. We monitored changes in other indices over the 12-month follow-up and compared them with baseline using repeated measures analysis of variance (ANOVA), followed by pairwise comparisons with Bonferroni correction for multiple testing. A mixed model for repeated measurements (MMRM) was used to manage assessments with missing data. A two-tailed *p* value of less than 0.025 was considered statistically significant for the 6-month HbA1c change, accounting for Bonferroni correction due to the dual primary endpoint design. For all other statistical tests, a significance level of 0.05 was applied. We conducted univariate analysis of patient characteristics predicting response in HbA1c greater than 0.3%. All statistical analyses were performed using SPSS version 25.0.

## Supplementary information

SUPPLEMENTAL MATERIAL

Protocol

## Data Availability

The datasets (including de-identified individual data) generated during the current study are available from the corresponding author upon request by contacting gjteng@seu.edu.cn, not for commercial use. All requests will be reviewed by the corresponding author and the sponsor, Shanghai Golden Leaf Med Tech (Brattea), within 2 weeks. A signed data access agreement with the sponsor is required before data sharing.
