# Supplementary material for: Catheter-based endovascular celiac and hepatic denervation for type 2 diabetes: a multicenter, open-label, single-arm study
Source: Signal Transduct Target Ther. 2025 Nov 13;10:371. doi: 10.1038/s41392-025-02459-6 (PMC12615629; doi:10.1038/s41392-025-02459-6)
Supplement: Supplementary file 1 — SUPPLEMENTAL MATERIAL [file 41392_2025_2459_MOESM1_ESM.docx]

Supplementary information

**Catheter-based endovascular celiac and hepatic denervation for type 2 diabetes: a multicenter, open-label, single-arm study**

**Contents**

Details of Celiac and Hepatic EDN Technique

*Supplementary Table 1*. Additional CGM measurements

*Supplementary Table 2.* Mean change of additional CGM measurements from baseline with 95% CI

*Supplementary Figure 1.* Results of OGTT.

**Celiac and Hepatic EDN Technique**

A surface electrode was placed on the back of the patient and connected to the console. With standard sterile technique, an 8 Fr sheath (Cordis, Waterloo, Belgium) was placed in the right femoral artery using Seldinger technique. Fluoroscopic guidance (Artis pheno, Siemens, Germany) was used throughout the procedure. For aortic angiography, a 5Fr pigtail catheter (Cordis, CA, USA) was placed over a 0.035-inch guidewire (Terumo, Japan). Then, a 5Fr Rh catheter (Cordis, CA, USA) and an 8Fr guiding catheter (Mach 1, Boston Scientific, MA, USA) were introduced coaxially for celiac and hepatic artery angiography and cannulation.

The guiding catheter was advanced over the guidewire and Rh catheter, positioned into the common hepatic artery, and a radiofrequency ablation catheter (Netrod, Brattea, China) with a hexapolar, basket-like design was introduced. After confirming the position, the guiding catheter was retracted, exposing the basket part of the ablation catheter. The retraction button was then used to pull the central wire, causing the basket to expand and adhere to the vessel wall. Before starting the first ablation, moderate sedation and analgesia with combinations of intravenous flurbiprofen and/or dezocine were administered and adjusted according to the patient's condition during the procedure. After confirming that at least 4 of the 6 electrodes were in solid contact with the arterial wall using the generator, an ablation session was performed with parameters of 60°C and 120 seconds, and impedance was monitored and limited up to 400Ω. During ablation, the catheter system monitored the temperature and impedance, altering radiofrequency energy delivery in response to a predetermined algorithm. Between each session, the basket was retracted by the button and slowly withdrawn within the artery. A total of at least 18 ablation sites were treated in the proper hepatic, common hepatic, and celiac arteries. Angiography was performed before, during, and after the EDN procedure to assess for any arterial abnormalities.

After the procedure, the puncture site was closed using a vascular closure device (ProGlide, Abbott, CA,USA), and the sheath introducer was removed. The puncture site was then appropriately compressed to ensure hemostasis, and the patient was transferred to the recovery ward for post-procedure monitoring and observation of vital signs.

**Supplementary Table 1. Additional CGM measurements**

|  | Baseline (n=30) | 1 month  (n=26) | 3 months (n=26) | 6 months (n=27) | 12 months (n=27) | *P* value |
| --- | --- | --- | --- | --- | --- | --- |
| Mean glucose (mmol/L) | 11.1 (2.1) | 9.8 (2.0) | 9.9 (1.9) | 10.5 (2.0) | 10.1 (1.9) | 0.002 |
| Mean overnight glucose (mmol/L) | 9.7 (2.0) | 8.4 (2.1) | 8.3 (1.8) | 9.1 (1.8) | 8.5 (2.0) | <0.001 |
| Time in range (3.9–10.0 mmol/L) | 42.6 (20.7) | 57.5 (22.5) | 56.5 (19.1) | 50.3 (21.2) | 54.3 (19.3) | 0.002 |
| Time in tight range (3.9–7.8 mmol/L) | 18.4 (16.2) | 33.9 (21.5) | 32.4 (18.4) | 26.7 (18.6) | 31.2 (19.6) | <0.001 |
| Time above range (> 10 mmol/L) | 56.8 (21.6) | 41.5 (23.7) | 42.2 (20.1) | 49.3 (21.6) | 44.7 (20.0) | 0.001 |
| Time above high range (> 13.9 mmol/L) | 24.1 (17.8) | 14.0 (12.7) | 15.6 (14.0) | 20.2 (14.5) | 16.2 (13.5) | 0.005 |
| Time below range (< 3.9 mmol/L) | 0.7 (1.6) | 1.1 (2.4) | 1.3 (2.6) | 0.5 (0.6) | 1.0 (1.4) | 0.304 |
| Coefficient of variation, % | 30.0 (4.9) | 30.8 (4.1) | 32.3 (5.4) | 30.3 (3.4) | 31.6 (4.8) | 0.099 |
| Standard deviation, mmol/L | 3.3 (0.6) | 3.0 (0.6) | 3.2 (0.7) | 3.2 (0.5) | 3.2 (0.6) | 0.057 |
| LAGE, mmol/L | 11.4 (1.7) | 10.9 (1.8) | 11.2 (1.9) | 11.2 (1.8) | 11.3 (1.7) | 0.460 |
| MAGE, mmol/L | 7.4 (1.3) | 6.9 (1.5) | 7.4 (1.4) | 7.5 (1.3) | 7.8 (1.6) | 0.002 |
| MODD, mmol/L | 2.8 (0.5) | 2.6 (0.6) | 2.7 (0.5) | 2.8 (0.5) | 2.8 (0.5) | 0.423 |

**Supplementary Table 2. Mean change of additional CGM measurements from baseline with 95% CI**

|  | 1 month  (n=26) | 3 months (n=26) | 6 months (n=27) | 12 months (n=27) |
| --- | --- | --- | --- | --- |
| Mean glucose (mmol/L) | -1.5 (-2.3—-0.7) | -1.4 (-2.3—-0.5) | -0.7 (-1.4—0.0) | -1.2 (-1.9—-0.4) |
| Mean overnight glucose (mmol/L) | -1.4 (-2.1—-0.7) | -1.6 (-2.5—-0.7) | -0.8 (-1.5—0.0) | -1.3 (-2.1—-0.5) |
| Time in range (3.9–10.0 mmol/L) | 15.4 (7.0—23.8) | 14.9 (6.1—23.6) | 7.6 (0.3—14.9) | 11.7 (4.2—19.2) |
| Time in tight range (3.9–7.8 mmol/L) | 15.9 (8.9—22.8) | 15.6 (8.1—23.0) | 7.8 (0.6—15.0) | 12.7 (5.0—20.4) |
| Time above range (> 10 mmol/L) | -15.8 (-24.7—-6.9) | -15.6 (-24.6—-6.5) | -15.2 (-15.2—-0.1) | -12.1 (-20.0—-4.1) |
| Time above high range (> 13.9 mmol/L) | -10.3 (-16.4—-4.2) | -9.3 (-16.6—-1.9) | -3.9 (-9.4—1.6) | -7.9 (-14.0—-1.7) |
| Time below range (< 3.9 mmol/L) | 0.4 (-0.9—1.6) | 0.7 (-0.2—1.6) | -0.2 (-0.9—0.5) | 0.4 (-0.5—1.2) |
| Coefficient of variation, % | 1.1 (-0.9—3.1) | 1.1 (-1.6—3.8) | 0.3 (-1.5—2.2) | 1.6 (-0.4—3.6) |
| Standard deviation, mmol/L | -0.4 (-0.6—-0.1) | -0.2 (-0.5—0.1) | -0.2 (-0.4—0.0) | -0.2 (-0.4—0.0) |
| LAGE, mmol/L | -0.5 (-1.1—0.1) | -0.3 (-1.0—0.3) | -0.3 (-0.9—0.4) | -0.1 (-0.8—0.5) |
| MAGE, mmol/L | -0.5 (-1.0—-0.1) | 0.0 (-0.4—0.4) | -0.1 (-0.9—0.6) | 0.4 (-0.1—0.9) |
| MODD, mmol/L | -0.2 (-0.4—0.0) | -0.1 (-0.3—0.2) | -0.1 (-0.4—0.1) | 0.0 (-0.3—0.3) |

LAGE, largest amplitude of glycemic excursions; MAGE, mean amplitude of glycemic excursions; MODD, mean of daily differences

Data are from post-hoc analyses. CGM=continuous glucose monitoring

*
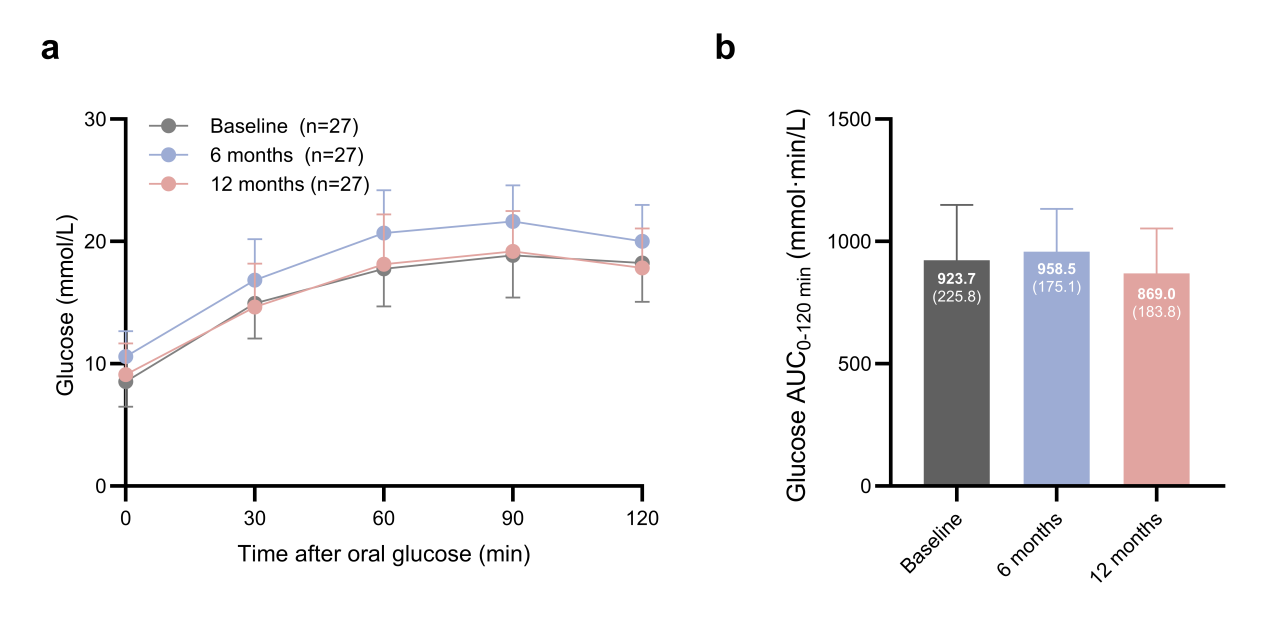
*

**Supplementary Fig.1. Results of OGTT.** (A) Plasma glucose concentrations over time (mean [SD]) post oral glucose tolerance test at baseline, 6 months and 12 months. (B) Glucose AUC_0-120 min_ post-OGTT
