# Supplementary material for: Catheter-based endovascular celiac and hepatic denervation for type 2 diabetes: a multicenter, open-label, single-arm study
Source: Signal Transduct Target Ther. 2025 Nov 13;10:371. doi: 10.1038/s41392-025-02459-6 (PMC12615629; doi:10.1038/s41392-025-02459-6)
Supplement: Supplementary file 2 — Protocol [file 41392_2025_2459_MOESM2_ESM.docx]

Protocol No. MLWY- S220501

**Clinical Trial Protocol for Medical Devices：**

**Endovascular Denervation for the Treatment of Type 2 Diabetes Mellitus**

**Name of the Medical Device:** Endovascular denervation system (the generator and the catheter)

**Model and Specification:**

generator: GL-06E

catheter:

| **Model** | **Specification** | **No. of electrodes** | **Length (mm)** | **Maximum expansion diameter (mm)** |
| --- | --- | --- | --- | --- |
| GL-6W | 08 | 6 | 1150±10% | 8 |
|  | 12 | 6 | 1150±10% | 12 |

**Protocol Version:** Version 1.3

**Document Date:** 09 Jan 2023

**Clinical Trial Institution:** Clinical Trial Institution of Zhongda Hospital Affiliated to Southeast University

**Leading Center/Coordinating Investigators:** Zhongda Hospital, Southeast University / Gaojun Teng, Ling Li

**Study Sponsor:** Shanghai Golden Leaf Med Tech (Brattea) Co., Ltd.

**CONFIDENTIAL**

The information contained in this document is the property of the sponsor. This document is provided in confidence. Without the prior written consent of the authorized representative of the sponsor, this document (in whole or in part), its copies or any proprietary information contained herein shall not be provided to any third party (except for the personnel within the research institution who assist in the work of this trial and are bound by a confidentiality agreement).

Version：MLWY- S220501

**Protocol Revision Record**

| **Version Number** | **Version Date** |
| --- | --- |
| V1.0 | July 20, 2022 |
| V1.1 | September 6, 2022 |
| V1.2 | September 22, 2022 |
| V1.3 | January 9, 2023 |

**CLINICAL STUDY PROTOCOL SYNOPSIS**

| **Protocol title** | Endovascular Denervation (EDN) for the Treatment of Type 2 Diabetes Mellitus |
| --- | --- |
| **Objective** | To evaluate the safety and efficacy of EDN at celiac and hepatic arteries in patients with type 2 diabetes who have inadequate glycemic control |
| **Study Design:** | This is a multicentre, open-label, single-arm intervention study with 37 patients with type 2 diabetes who have suboptimal or poor glycemic control (i.e., glycated haemoglobin (HbA1c >7.5% and <10.5%) on two to four antidiabetic drugs, including metformin)  The study has 4 distinct stages, as follows.  **Stage 1:** Screening  HbA1c will be confirmed during screening along with additional parameters of eligibility  **Stage 2:** Run-in period  A 14-day run-in period will be included to conduct continuous glucose monitoring (CGM) and collect other parameters.  **Stage 3:** Treatment  Each patient will undergo EDN. Discrete radiofrequency ablations at 60 ℃, each lasting up to 120 seconds will be applied to obtain at least 18 ablations, with a maximum of up to 6 electrodes delivering energy simultaneously during each session. Ablations will be performed at the celiac, common hepatic and proper hepatic arteries  **Stage 4:** Follow-up  Patients will be followed up at 1, 3, 6, 12, and 24 months for AEs, laboratory tests and other parameters |
| **Data Management** | The data management of this trial will be undertaken by a Contract Research Organization (CRO). The data management process shall comply with the relevant regulatory requirements for data management and follow the standard operating procedures (SOPs) of the data management department to ensure the authenticity, accuracy, integrity, and reliability of the clinical trial data. |
| **Number of Subjects**  **Planned** | Approximately 30 patients will be treated with EDN. Replacement subjects may be added if subjects are screen failures or withdraw prior to drug product infusion. |
| **Inclusion Criteria** | - Age of 18–65 years old (both inclusive) - Understand the requirements and treatments of the trial, agreed to and were able to complete all follow-up assessments required for the trial, and signed informed consent before any special trial-related tests and treatments were performed - Diagnosed with T2DM for ≥1 and <15 years (according to WHO criteria) - Metformin (daily dose ≥1000mg) was combined with 1-3 oral antidiabetic drugs (OADs) for more than 3 months and/or insulin (no dose limit). Specific OADs were: insulin secretagogues (sulfonylureas/glinides), thiazolidinediones and α-glucosidase inhibitors, and the combined OADs were at least half of the maximum approved dose in the package insert - Glycosylated hemoglobin of 7.5–10.5% (58–91 mmol/mol) (both inclusive) - Body mass index of 18–40 kg/m^2^ (both inclusive) |
| **Exclusion Criteria** | - Type 1 diabetes, late-onset autoimmune diabetes in adults (LADA), or any secondary diabetes. - Previous aortic disease (e.g., aortic aneurysm or dissection) or aortic surgery (including celiac artery denervation). - Baseline CTA showing aortic aneurysm or dissection, anatomical abnormalities of the hepatic artery and its branches, or other abnormal vascular structure/status (e.g., severe tortuosity or stenosis of the artery, intravascular thrombus, or unstable plaque) deemed unsuitable for vascular ablation by the investigator. - More than two self-reported or documented episodes of severe hypoglycemia in the past 6 months (defined as hypoglycemia with severe cognitive impairment requiring assistance from another person). - More than one documented episode of hyperglycemia requiring hospitalization in the past 6 months, including diabetic ketoacidosis or hyperosmolar coma. - Severe diabetic complications, such as retinal, renal, vascular, neuropathy, or diabetic foot, deemed ineligible for enrollment by the investigator. - Major cardiovascular and cerebrovascular events (MACCE) within the past 6 months, including cerebrovascular accident (CVA), transient cerebral ischemia (TIA), heart failure (NYHA class III-IV), acute myocardial infarction, or unstable angina requiring hospitalization (including previous coronary artery bypass grafting or coronary stent implantation); and uncontrolled or severe arrhythmias. - Severe autonomic neuropathy (orthostatic hypotension, gastroparesis syndrome, etc.). - Untreated or uncontrolled high blood pressure (SBP ≥ 160 mmHg or DBP ≥ 100 mmHg), or low blood pressure (BP < 90/50 mmHg). - History of renal insufficiency or failure with baseline estimated glomerular filtration rate (eGFR) < 60 mL/min/1.73 m² (see Note C of the Clinical Data Collection Table in Table 5-2 for the eGFR formula). - Chronic active hepatitis, severe hepatobiliary disease (including cirrhosis), or hepatic insufficiency (alanine and/or aspartate aminotransferase > 3 times the upper limit of normal or serum total bilirubin > 2 times the upper limit of normal). - Acute or chronic pancreatitis during screening and baseline periods. - Acute systemic infections during screening and baseline periods. - Bleeding tendency or coagulopathy (PT, APTT, or INR > 2 times the upper limit of normal; platelet count < 80×10⁹/L or ≥ 700×10⁹/L). - Active GI ulcer or GI bleeding within 3 months before baseline. - Symptomatic cholelithiasis (including cholecystolithiasis, extrahepatic bile duct stones, and intrahepatic bile duct stones) without effective treatment such as cholecystectomy, choledocholithotomy, internal drainage, or external drainage. - Hyperthyroidism, hypothyroidism, acromegaly, Cushing's syndrome, or other endocrine and metabolic diseases. - Autoimmune diseases. - Diagnosed with a high risk of malignancy or cancer development/recurrence, or expected life expectancy < 12 months. - Major surgical procedures within the past 3 months. - Conditions or concomitant medical conditions, such as hemoglobinopathy or hemolytic anemia, preventing the primary endpoint from being assessed. - Mental illness preventing cooperation. - Treatment with a GLP-1 receptor agonist, DPP-4 inhibitor, or SGLT-2 inhibitor within 3 months before the screening period. - Systemic or intra-articular glucocorticoids (other than topical, inhaled, or eye drops) within 3 months before the screening period. - Use of weight-loss medications such as orlistat or other treatments for weight loss (weight-loss tea/herbal medicine/acupuncture, etc.) within 3 months before the screening period. - Prior or anticipated bariatric surgery such as subtotal gastrectomy or liposuction. - Use of central sympathetic inhibitors or anticonvulsants within 3 months prior to or anticipated during the screening period. - Long-term anticoagulation therapy requiring preoperative heparin bridging anticoagulation that is not possible. - Weight gain of more than 10% in the past 3 months. - Allergy to or contraindication to contrast media, with inadequate pretreatment as judged by the investigator. - Known history of allergy to the study device (containing polytetrafluoroethylene or Nitinol) or medications associated with the trial protocol. - Participation in other clinical studies within 3 months before enrollment. - Anticipation of participation in a clinical trial of another drug or medical device within 24 months after baseline surgery. - Pregnancy or lactation, or plans to become pregnant within the next 2 years (all women of childbearing age must undergo a pregnancy test within 7 days before baseline surgery). - Drastic changes in diet and exercise habits expected during the study (due to religious/work needs/weight loss, etc.). - Irregular day and night work (night shift workers). - History of alcohol, drug, or substance abuse. - Scheduled periodic blood product therapy or severe blood loss during the previous 3 months/study period. - Any condition, as judged by the investigator, that affects the safety of the participant or interferes with the evaluation of the test results. - Aortic aneurysm or aortic dissection confirmed on angiography before the procedure.   Vascular structure and conditions deemed unsuitable for ablation (e.g., severe tortuosity or stenosis of the artery, abnormal vascular anatomy, thrombus, or unstable plaque). |
| **Endpoints** | **Primary Endpoints:**   - The occurrence of composite major adverse events (MAE) related to the device and/or the procedure within 30 days post-procedure. MAE includes the following events:  1. Hypoglycemic or hyperglycemic events requiring hospitalization 2. Vascular complications requiring surgical repair, interventional treatment, thrombin injection, or blood transfusion 3. Celiac/hepatic artery dissection or perforation requiring intervention 4. Significant embolic events leading to end-organ damage or requiring intervention to prevent end-organ damage 5. Acute liver dysfunction or liver failure 6. All-cause mortality  - The change in glycated hemoglobin (HbA1c) at 6 months post-procedure compared to baseline levels.   **Secondary endpoints:**   - The changes of Hba1c from baseline at 1, 3, 12 and 24 months post procedure - The changes of assessment of β-cell function and insulin resistance (Homeostatic Model Assessment for β-cell function and insulin resistance, HOMA-β/HOMA-IR) - The changes of mean glucose, postprandial glucose increase, nocturnal glucose increase and time in range (TIR, 3.9-10.3 mmol/L) by 14 - day continuous glucose monitoring (CGM) - The changes of blood lipids (triglycerides, total cholesterol, high- and low-density lipoprotein) - The changes of liver function (alanine aminotransferase, aspartate aminotransferase, alkaline phosphatase and gamma - glutamyl transferase) - The changes of renal function (urea nitrogen, blood creatinine and eGFR) - The changes of glucose, insulin and C - peptide in oral glucose tolerance test (OGTT) - Proportion of patients achieving target HbA1c (< 7.0% and ≤6.5%) - Proportion of patients with HbA1c < 7% without hypoglycemia/severe hypoglycemia or weight gain - Proportion of patients with the changes of in type/dose of antidiabetic drugs - Incidence of hypoglycemia and severe hypoglycemia (blood glucose level < 3.9 mmol/L) - The occurrence of new significant celiac or hepatic artery stenosis at 6 months post procedure (stenosis >50% as assessed by CT angiography and confirmed by digital subtrationsubtraction angiography when CT angiography assessment shows abnormal) - The occurrence of device and/or procedure-related adverse events and serious adverse events - Incidence of cardiovascular and cerebrovascular complications (defined as cardiovascular death, myocardial infarction and ischemic stroke) - Quality of life assessment (EQ-5D-5L) |
| **Statistical Methods** | **Sample size estimation:**  No formal sample size calculation was performed. However, the sample size of 30 (treated) was determined based on the hypothesis that patients would experience a reduction in HbA1c at 6 months. To achieve a predefined 1.0% reduction in HbA1c, with a standard deviation of 1.5%, a sample size of 26 would be required to yield a lower two-sided 95% confidence bound of approximately 0. Taking into account a 10% drop out rate, the estimated sample size is 29.  **Populations for analysis:**  Baseline characteristics were analyzed in all enrolled patients. The treated patients were analyzed for safety profile, and patients with follow-ups were analyzed for efficacy.  **Analysis of primary endpoints:**  All adverse events were recorded by and assessed by all investigators for the association with the device or procedure. HbA1c reductions at 6 months were measured for patients with 6-month follow-up and expressed as mean with a 2-sided 95% CI.  The success criterion for this study is defined as no device- or procedure-related MAE occured and achievement of HbA1c reduction at 6 months. |

**Protocol Signature Page**

**Protocol Title:** Endovascular Denervation for the Treatment of Type 2 Diabetes – Clinical Trial Protocol

**Declaration of the Statistical Analysis Unit:**

I have read this protocol. The study will be conducted in accordance with the ethical, moral, and scientific principles set forth in the Declaration of Helsinki (2013) and the Good Clinical Practice for Medical Device Clinical Trials in China (2022). I agree to conduct this clinical trial in compliance with the protocol design and requirements.

During the course of the study, I will strictly adhere to the requirements of this protocol. Any modification to the protocol may only be implemented after notification and approval by the Sponsor, and upon re-approval or record-filing by the Ethics Committee, unless immediate measures are necessary to protect the safety, rights, and interests of the subjects.

I will keep this protocol and all related information confidential.

Name of the Statistical Analysis Unit:

Peking University Clinical Research Institute

Signature of the Responsible Statistician: _________________________

Date: _________________________

**Protocol Signature Page**

**Protocol Title:** Endovascular Denervation for the Treatment of Type 2 Diabetes – Clinical Trial Protocol

**Declaration of the Data Management Unit:**
I have read this protocol. The study will be conducted in accordance with the ethical, moral, and scientific principles set forth in the Declaration of Helsinki (2013) and the Good Clinical Practice for Medical Device Clinical Trials in China (2022). I agree to conduct this clinical trial in compliance with the protocol design and requirements.

During the course of the study, I will strictly adhere to the requirements of this protocol. Any modification to the protocol may only be implemented after notification and approval by the Sponsor, and upon re-approval or record-filing by the Ethics Committee, unless immediate measures are necessary to protect the safety, rights, and interests of the subjects.

I will keep this protocol and all related information confidential.

Name of the Data Management Unit:
Beijing Giant Med-Pharma Services, Inc.

Signature of the Responsible Statistician: _________________________

Date: _________________________

Table of Contents

[Abbreviations 5](#_Toc1853)

[Protocol Synopsis 6](#_Toc15557)

[1. Sponsor Information 16](#_Toc16800)

[2. Participating Information 16](#_Toc27497)

[2.1 List of Clinical Trial Institutions and Investigators 16](#_Toc2802)

[2.2 Statistical Analysis Unit Information 16](#_Toc17365)

[2.3 CRO Information 16](#_Toc18465)

[3. Background Information of the Clinical Trial 16](#_Toc3602)

[3.1 Current Status of T2DM 16](#_Toc25779)

[3.2 Research Background of Endovascular Denervation (EDN) for T2DM 18](#_Toc17569)

[3.3 Introduction of the Investigational Device 20](#_Toc8666)

[3.3.1 Device Characteristics 20](#_Toc23001)

[3.3.2 Structure, Working Principle, and Mechanism of Action of the Endovascular Denervation System 21](#_Toc11981)

[3.4 Scope of Application and Related Information 36](#_Toc13645)

[3.4.1 Trial Scope 36](#_Toc23849)

[3.4.2 Indications 36](#_Toc4402)

[3.4.3 Contraindications 36](#_Toc6447)

[3.4.4 Precautions 37](#_Toc24604)

[4. Study Objective 39](#_Toc19735)

[5. Study Design 39](#_Toc26002)

[5.1 Overall Design and Rationale 39](#_Toc20431)

[5.2 Subject Selection 40](#_Toc16449)

[5.2.1 Inclusion Criteria 40](#_Toc17118)

[5.2.2 Exclusion Criteria 41](#_Toc23262)

[5.2.3 Subject Withdrawal Criteria and Procedures 45](#_Toc8265)

[5.3 Evaluation Methods 46](#_Toc8434)

[5.3.1 Primary Endpoints 46](#_Toc168)

[5.3.2 Secondary Endpoints 47](#_Toc32548)

[5.3.3 Additional Assessments 48](#_Toc8905)

[5.4 Study Flow 49](#_Toc12991)

[5.4.1 Study Flowchart 49](#_Toc18842)

[5.4.2 Data Collection 51](#_Toc19081)

[5.4.3 Treatment Assignment 60](#_Toc27180)

[5.4.4 Subject Screening 60](#_Toc2850)

[5.4.5 Informed Consent 61](#_Toc13073)

[5.4.6 Screening Period 61](#_Toc15234)

[5.4.7 Baseline Period (within 14 days pre-procedure) 63](#_Toc32610)

[5.4.8 Enrollment and Baseline Procedure 68](#_Toc16983)

[5.4.9 Post-op Day 7 or Before Discharge 69](#_Toc6955)

[5.4.10 30-Day Follow-up (±7 days) 69](#_Toc839)

[5.4.11 3-Month Follow-up (±14 days) 70](#_Toc11287)

[5.4.12 6-Month Follow-up (±30 days) 70](#_Toc9069)

[5.4.13 12-Month Follow-up (±30 days) 71](#_Toc26762)

[5.4.14 24-Month Follow-up (±30 days) 71](#_Toc22149)

[5.4.15 End of Study 71](#_Toc22674)

[5.4.16 Source Documents 72](#_Toc8862)

[5.5 Device Use Specifications 72](#_Toc30548)

[5.5.1 Surgical Technique 72](#_Toc13907)

[5.5.2 Surgical Workflow 72](#_Toc221)

[6. Statistical Considerations 74](#_Toc3548)

[6.1 Primary Endpoints 74](#_Toc22398)

[6.2 Study Hypothesis 75](#_Toc17768)

[6.3 Sample Size 76](#_Toc1318)

[6.4 Statistical Methods 76](#_Toc11180)

[7. Monitoring Plan 77](#_Toc16459)

[8. Data Management 78](#_Toc32139)

[8.1 Data Entry 78](#_Toc22885)

[8.2 Database Setup and Data Verification 78](#_Toc15415)

[8.3 Database Lock 79](#_Toc23823)

[8.4 Data Processing 79](#_Toc22858)

[9. Risk–Benefit Analysis 80](#_Toc22591)

[9.1 Risks and Adverse Events Related to Endovascular Procedures 80](#_Toc4254)

[9.2 Potential Risks Related to the Investigational Device and EDN 81](#_Toc22329)

[9.3 Study-Related Risks 82](#_Toc17903)

[9.4 Risk Minimization Measures 82](#_Toc15658)

[9.5 Expected Benefits 82](#_Toc11407)

[9.6 Risk–Benefit Ratio 83](#_Toc16784)

[10. Quality Control of the Clinical Trial 83](#_Toc29104)

[11. Ethical Considerations and Informed Consent 85](#_Toc2515)

[11.1 Ethical Considerations 85](#_Toc9750)

[11.2 Protocol Approval 86](#_Toc18621)

[11.3 Informed Consent Process and ICF Document 86](#_Toc18982)

[12. Requirements for Reporting Adverse Events and Device Deficiencies 88](#_Toc23229)

[12.1 Definitions of Adverse Events 88](#_Toc14261)

[12.2 Reporting of Adverse Events 91](#_Toc19722)

[12.3 Causality Assessment with Investigational Device/Procedure 92](#_Toc16129)

[12.4 Investigator Reporting Requirements 94](#_Toc28458)

[12.5 Device Deficiencies 96](#_Toc14518)

[12.6 Sponsor Reporting Requirements 97](#_Toc18793)

[13. Responsibilities of the Parties 98](#_Toc7417)

[13.1 Responsibilities of the Investigator 98](#_Toc26686)

[13.2 Ethics Committee (EC) 101](#_Toc28608)

[13.3 Responsibilities of the Sponsor 101](#_Toc11192)

[14. Insurance 102](#_Toc5384)

[15. Statement of Compliance 102](#_Toc866)

[16. Committees 103](#_Toc29609)

[16.1 Executive Committee 103](#_Toc579)

[16.2 Safety Oversight Process 103](#_Toc17366)

[17. Study Suspension or Termination 103](#_Toc21479)

[17.1 Early Termination by the Sponsor 103](#_Toc3723)

[17.2 Termination by Investigator/Site or EC Withdrawal 104](#_Toc8843)

[17.3 Criteria for Suspending/Terminating Site Participation 105](#_Toc19829)

[18. Publication Policy 105](#_Toc16363)

[19. References 105](#_Toc22298)

# Abbreviations

| **Abbreviations** | **Definition** |
| --- | --- |
| ADE | Adverse Device Effect |
| AE | Adverse Event |
| BMI | Body Mass Index |
| BSA | Body Surface Area |
| CRF | Case Report Form |
| CRO | Contract Research Organization |
| DSA | Digital Subtraction Angiography |
| ECG | Electrocardiography |
| eCRF | Electronic Case Report Form |
| EDC | Electronic Data Capture System |
| EDN | Endovascular Denervation |
| HbA1c | Hemoglobin A1C |
| IR | Insulin Resistance |
| MAE | Major Adverse Event |
| NMPA | National Medical Products Administration |
| OAD | Oral Antidiabetic Drug |
| OGTT | Oral Glucose Tolerance Test |
| QF | Query Form |
| RDN | Renal Denervation |
| SAE | Serious Adverse Event |
| SADE | Serious Adverse Device Effect |
| SBP | Systolic Blood Pressure |
| T2DM | Type 2 Diabetes Mellitus |

# Protocol Synopsis

| **Endovascular Denervation for the Treatment of Type 2 Diabetes** | |
| --- | --- |
| **Objective** | To evaluate the safety and efficacy of the Endovascular Denervation System (including generator and catheter) manufactured by Shanghai Golden Leaf Med Tech (Brattea) Co., Ltd., when performing endovascular denervation (EDN) in the celiac trunk and hepatic artery for the treatment of type 2 diabetes mellitus (T2DM). |
| **Device** | Endovascular Denervation Generator and Catheter |
| **Control Device** | None |
| **Device Specifications** | - Endovascular Denervation Catheter  \| **Model** \| **Specification** \| **Number of Electrodes** \| **Stent Type** \| **Length (mm)** \| **Maximum Expansion Diameter (mm)** \| \| --- \| --- \| --- \| --- \| --- \| --- \| \| GL-6W \| 08 \| 6 \| Net \| 1150±10% \| 8 \| \| 12 \| 6 \| Net \| 1150±10% \| 12 \|  - Endovascular Denervation Generator: Model: GL-06E (6×15 W, temperature-controlled, 12.1-inch LCD resistive touchscreen display) |
| **Study Design** | Prospective, multicenter, single-arm feasibility clinical trial |
| **Study Population** | Patients with poorly controlled T2DM, defined as those who, despite treatment with metformin in combination with one to three oral antidiabetic drugs (OADs) and/or insulin, still fail to achieve adequate glycemic control, with HbA1c levels between 7.5% and 10.5%. |
| **Planned Sample Size** | 30 |
| **Planned Participating Hospitals** | Zhongda Hospital, Southeast University  The First Affiliated Hospital of University of Science and Technology of China (Anhui Provincial Hospital)  Shandong Provincial Hospital |
| **Primary Endpoints** | Primary Safety Endpoint: Incidence of composite major adverse events (MAEs) related to the investigational device and/or ablation procedure occurring intraoperatively or within 30 days post-procedure.  MAEs include:   - Hypoglycemia or hyperglycemia events requiring hospitalization - Vascular complications requiring surgical repair, interventional therapy, thrombin injection, or blood transfusion - Celiac/hepatic artery dissection or perforation requiring intervention - Significant embolic events resulting in, or requiring intervention to prevent, end-organ damage - Acute liver injury or hepatic failure - All-cause death   Primary Efficacy Endpoint: Change in HbA1c from baseline to 6 months post-procedure. |
| **Secondary Endpoints** | The following endpoints will be evaluated at 1, 3, 6, 12, and 24 months post-procedure:   1. Changes from baseline at 1, 3, 6, 12, and 24 months in the following parameters:  - HbA1c (at 1, 3, 12, and 24 months) - Assessment of β-cell function and insulin resistance (including HOMA-β and HOMA-IR, formulas provided in Table 5-2, footnote f) - Continuous glucose monitoring (14-day) outcomes: mean glucose, postprandial excursions, nocturnal excursions, and time-in-range (TIR), measured using non-invasive CGM - Lipid profile (triglycerides, total cholesterol, HDL-C, LDL-C) - Liver function tests (ALT, AST, ALP, GGT) - Renal function tests (BUN, serum creatinine, and eGFR, formula provided in Table 5-2, footnote c)  1. Oral glucose tolerance test (OGTT) and insulin and C-peptide release test (5-point sampling at 0, 30, 60, 90, 120 minutes): changes from baseline at 6, 12, and 24 months. 2. Proportion of patients achieving HbA1c targets (<7.0% and ≤6.5%). 3. Composite endpoint: proportion of patients achieving HbA1c <7.0% without hypoglycemia/severe hypoglycemia or weight gain 4. Changes in types/dosages of antidiabetic medications and proportion of patients with dose reduction. 5. Incidence of hypoglycemia and severe hypoglycemia (blood glucose <3.9 mmol/L). 6. Incidence of new-onset significant stenosis (>50%) of the celiac trunk, hepatic artery, or branches at 6 months post-procedure, confirmed by digital subtraction angiography (DSA) in cases with abnormal CTA findings. 7. Incidence of adverse events (AEs) and serious adverse events (SAEs) related to the investigational device and/or baseline procedure, occurring during the baseline procedure, 7 days/discharge, and at 1, 3, 6, 12, and 24 months post-procedure. 8. Incidence of major cardiovascular and cerebrovascular events (3MACE) at 12 and 24 months, defined as cardiovascular death, myocardial infarction, and ischemic stroke. 9. Quality of life and lifestyle assessment at baseline and 6 months post-procedure, including the EuroQol 5-Dimension 5-Level Questionnaire (EQ-5D-5L). |
| **Additional Exploratory Endpoints** | - Body weight and BMI - Waist circumference and waist-to-hip ratio - Office blood pressure - Heart rate variability (Holter) - Catecholamine levels (epinephrine, norepinephrine) - Device performance evaluation (investigator feedback) |
| **Enrollment Criteria** | Subjects who sign IRB-approved informed consent and meet all inclusion criteria without exclusion criteria will be enrolled. Enrollment is defined at the time of catheter insertion. |
| **Follow-up Schedule** | Clinical follow-ups at: 7 days or discharge, 30 days (±7), 3 months (±14), 6 months (±30), 12 months (±30), 24 months (±30). |
| **Study Duration** | Enrollment: 3 months; Follow-up: 24 months; Total: 27 months. |
| **Concomitant Antidiabetic Therapy & Lifestyle Management** | - Stable OAD treatment ≥3 months prior to baseline required (with 2-week washout for α-glucosidase inhibitors). - Post-EDN, OADs and insulin may be adjusted per guidelines based on glucose control. - Lifestyle counseling (diet, exercise, hypoglycemia education) will be provided throughout. - Rescue therapy: insulin adjustment (max 0.5–0.6 U/kg/day). - Hypoglycemia management: 15–20 g glucose, repeat testing at 15 minutes, and stepwise drug reduction (insulin → sulfonylureas → glinides → TZDs → metformin). - Lifestyle: maintain consistent exercise and diet; no new structured weight-loss programs allowed. |
| **Inclusion Criteria** | - Age 18–65 years - Able to understand study requirements and sign informed consent - T2DM diagnosed 1–15 years (WHO criteria) - On metformin (≥1000 mg/day) + 1–3 OADs and/or insulin for ≥3 months, with OAD dose ≥50% of maximum approved dose - HbA1c 7.5–10.5% at baseline - BMI 18–40 kg/m²   (α-glucosidase inhibitors require 2-week washout before enrollment) |
| **Exclusion Criteria** | **Subjects with any of the following medical histories or evidence*:**   1. Type 1 diabetes mellitus, latent autoimmune diabetes in adults (LADA), or any form of secondary diabetes. 2. History of aortic disease (e.g., aneurysm or dissection) or prior aortic surgery (including celiac denervation). 3. Baseline CTA showing aortic aneurysm or dissection, hepatic artery or branch anatomical abnormalities, or other vascular structures/conditions unsuitable for ablation as judged by the investigator (e.g., severe tortuosity or stenosis, intraluminal thrombus, unstable plaque). 4. More than two documented or self-reported episodes of severe hypoglycemia (defined as hypoglycemia with severe cognitive impairment requiring assistance) within the past 6 months. 5. More than one documented hyperglycemic crisis requiring hospitalization within the past 6 months, including diabetic ketoacidosis or hyperosmolar coma. 6. Severe diabetic complications (retinopathy, nephropathy, vasculopathy, neuropathy, diabetic foot) deemed unsuitable for study participation by the investigator. 7. Major adverse cardiovascular or cerebrovascular event (MACCE) within the past 6 months, including cerebrovascular accident (CVA), transient ischemic attack (TIA), heart failure (NYHA class III–IV), acute myocardial infarction, or unstable angina requiring hospitalization (including CABG or PCI), or uncontrolled/severe arrhythmia. 8. Severe autonomic neuropathy (e.g., orthostatic hypotension, gastroparesis). 9. Untreated or uncontrolled severe hypertension (SBP ≥160 mmHg or DBP ≥100 mmHg), or hypotension (BP <90/50 mmHg). 10. History of renal insufficiency or renal failure, with baseline eGFR <60 mL/min/1.73 m² (see Table 5-2, footnote c). 11. Chronic active hepatitis, severe hepatobiliary disease (including cirrhosis), or hepatic failure (ALT/AST >3× ULN or total bilirubin >2× ULN). 12. Acute or chronic pancreatitis during screening or baseline. 13. Acute systemic infection during screening or baseline. 14. History of bleeding tendency or coagulopathy (PT, APTT, or INR >2× ULN; platelet count <80×10⁹/L or ≥700×10⁹/L). 15. Active peptic ulcer or GI bleeding within 3 months prior to baseline. 16. Symptomatic cholelithiasis (gallstones) without effective treatment (e.g., cholecystectomy, bile duct stone removal, drainage). 17. Other endocrine/metabolic disorders (e.g., hyperthyroidism, hypothyroidism, acromegaly, Cushing’s syndrome). 18. Autoimmune diseases. 19. Malignancy, high risk of recurrence, or life expectancy <12 months. 20. Major surgery within 3 months prior to baseline. 21. Hemoglobinopathy, hemolytic anemia, or other conditions that may interfere with endpoint evaluation. 22. Psychiatric illness that impairs compliance.   *Note: Patients failing inclusion criteria during screening may be rescreened after investigator assessment.  **Prohibited Prior or Concomitant Therapies**   1. GLP-1 receptor agonists, DPP-4 inhibitors, or SGLT-2 inhibitors within 3 months prior to screening. 2. Systemic or intra-articular corticosteroid therapy within 3 months prior to screening (topical, inhaled, or ophthalmic excluded). 3. Weight-loss drugs (e.g., orlistat) or other weight-reducing interventions (e.g., slimming tea, herbal medicine, acupuncture) within 3 months prior to screening. 4. Prior or planned bariatric procedures (e.g., gastrectomy, liposuction). 5. Central sympatholytic or anticonvulsant medications within 3 months prior to screening or anticipated during the study. 6. Requirement for long-term anticoagulation in patients unsuitable for peri-procedural heparin bridging.   **Other Exclusion Criteria**   1. Body weight change >10% within 3 months prior to screening. 2. Known allergy or contraindication to contrast agents, unmanageable by premedication. 3. Known allergy to investigational device components (e.g., PTFE, nitinol) or study-related drugs. 4. Participation in another clinical study within 3 months prior to enrollment or concurrent participation in any clinical trial. 5. Anticipated participation in another drug or device trial within 24 months post-procedure. 6. Pregnant or breastfeeding women, or women planning pregnancy within 2 years (all women of childbearing potential must have a negative pregnancy test within 7 days prior to baseline procedure). 7. Anticipated major changes in diet or physical activity (e.g., due to religion, occupation, or weight-loss programs). 8. Irregular circadian rhythm (e.g., night-shift workers). 9. History of alcoholism, drug abuse, or substance misuse. 10. Receipt of blood products or severe blood loss within 3 months prior to screening, or anticipated need for repeated transfusions during study. 11. Any condition deemed by the investigator to compromise subject safety or interfere with study assessments.   **Angiographic Exclusion Criteria**   1. Aortic aneurysm or dissection confirmed on angiography prior to EDN. 2. Vascular anatomy or condition unsuitable for ablation as judged by the investigator (e.g., severe tortuosity, stenosis, anatomical abnormalities, intraluminal thrombus, unstable plaque). |
| **Statistical Considerations** | |
| **Primary Statistical Hypothesis** | This is a single-arm feasibility trial with no formal statistical hypothesis. |
| **Statistical Methods** | Descriptive statistics will be applied for baseline demographics, safety endpoints, and categorical data. Paired t-tests will be used for efficacy endpoints (e.g., change in HbA1c and other variables from baseline to post-procedure). One-sided 97.5% CI lower limits of mean changes will be calculated |
| **Sample Size** | A total of 30 subjects will be enrolled to support feasibility objectives. |

# Sponsor Information

| **Name** | **Address** | **Contact Person** | **Contact Information** |
| --- | --- | --- | --- |
| Shanghai Golden Leaf Med Tech (Brattea) Co., Ltd. | 4th Floor, Building 2, No. 466 Yindu Road, Xuhui District, Shanghai, China | Chunni Zhu | - |

# Participating Information

## List of Clinical Trial Institutions and Investigators

| **Site Code** | **Institution Name** | **Investigator** | **Title** | **Contact Information** |
| --- | --- | --- | --- | --- |
| 1 | Zhongda Hospital, Southeast University | Gao-Jun Teng | Chief Physician | - |
| 1 | Zhongda Hospital, Southeast University | Ling Li | Chief Physician | - |
| 2 | The First Affiliated Hospital of USTC (Anhui Provincial Hospital) | Jianping Weng | Chief Physician | - |
| 3 | Shandong Provincial Hospital | Jiajun Zhao | Chief Physician | - |

## Statistical Analysis Unit Information

| **Statistical Unit Name** | **Address** | **Contact Person** | **Contact Information** |
| --- | --- | --- | --- |
| Peking University Clinical Research Institute | No. 38 Xueyuan Road, Haidian District, Beijing | Chen Yao | - |

## CRO Information

| **Name** | **Address** | **Contact Person** | **Contact Information** |
| --- | --- | --- | --- |
| Beijing Giant Med-Pharma Services, Inc. | Room 2002, Jincheng Jianguo No. 5 Building, No. 5 Jianguomen North Street, Dongcheng District, Beijing | Kaiwen Liu | - |

# Background Information of the Clinical Trial

## 3.1 Current Status of T2DM

According to the latest statistics from the International Diabetes Federation (IDF), there are currently about 537 million adults (aged 20–79 years), representing approximately 10% of the global population, living with diabetes, and the prevalence is increasing year by year. In 2021, diabetes-related deaths reached 6.7 million, accounting for 12.2% of all-cause deaths in the 20–79 age group. ^1^ China, as the most populous country in the world, also has the largest number of people with diabetes. Data from 2021 show that there were approximately 140 million patients with diabetes in China, with 1.4 million diabetes-related deaths. More than 90% of these cases were T2DM.^1-3^ Because T2DM is often asymptomatic in its early stages, diagnosis is usually delayed, and the actual prevalence is likely higher than currently reported. Untreated or uncontrolled diabetes significantly increases the risk of complications such as cardiovascular, cerebrovascular, renal, retinal, and neurological diseases, as well as disability.^3,4^ Diabetes imposes a substantial economic burden on individuals and society. It is estimated that global direct medical expenditures on diabetes exceed USD 966 billion annually, representing 11.2% of global health expenditure. This figure has increased by 316% over the past 15 years, and the projected GDP loss between 2011 and 2030 exceeds USD 1.7 trillion.^1^

T2DM is a metabolic disease characterized by chronic hyperglycemia caused by insulin resistance and varying degrees of β-cell dysfunction.^3,5^ Patients with T2DM often present with one or more components of metabolic syndrome, such as hypertension, dyslipidemia, and obesity. With worsening hyperglycemia, hypertension, dyslipidemia, and weight gain, the risk, progression, and severity of complications increase significantly. Therefore, treatment strategies for T2DM must be comprehensive, including glucose-lowering, antihypertensive, lipid-regulating, antithrombotic, weight control, and lifestyle modifications.^2^ Glycemic control is central to the management of T2DM. HbA1c is the key clinical indicator reflecting long-term glucose control and is closely associated with the risk of diabetic complications. Previous clinical studies have shown that each 1% reduction in HbA1c reduces the risk of all diabetes-related endpoints and diabetes-related mortality by 21% (*P* < 0.01), myocardial infarction by 14% (*P* < 0.01), and microvascular complications by 37% (*P* < 0.01).^6,7^ Furthermore, early intensive glycemic control provides long-term benefits. Consequently, current guidelines recommend a target HbA1c <7% for most non-pregnant adults with T2DM.^2,8^

However, T2DM is a lifelong disease, and during long-term treatment, poor patient adherence is common due to difficulties in sustaining lifestyle changes and medication compliance. Even among adherent patients, drug efficacy may decline over time, making glycemic control unsatisfactory and adversely affecting prognosis.^9,10^ Hence, exploring alternative treatments beyond glucose-lowering drugs has significant clinical importance.

## 3.2 Research Background of Endovascular Denervation (EDN) for T2DM

The autonomic nervous system regulates and maintains energy metabolism balance through sympathetic and parasympathetic innervation of target organs. Sympathetic overactivity reduces glucose uptake, suppresses muscle cell function, and induces insulin resistance via hemodynamic and metabolic effects. Multiple studies have demonstrated that sympathetic hyperactivity is strongly associated with insulin resistance and T2DM, contributing to fasting hyperglycemia, hypertension, obesity, and other metabolic abnormalities.^11-13^ Compared with healthy individuals, T2DM patients show markedly elevated sympathetic activity.^14^ A positive feedback loop exists between sympathetic overactivity and insulin resistance, creating a vicious cycle.^15^ Therapeutic strategies targeting sympathetic overactivity may disrupt this cycle and restore metabolic balance in T2DM

Percutaneous renal denervation (RDN) has been developed to treat resistant hypertension by delivering radiofrequency (RF) energy within the renal arteries to ablate surrounding sympathetic nerves.^16,17^ Early RDN studies reported improvements in glucose metabolism and insulin sensitivity in hypertensive patients with comorbid T2DM.^18-20^ However, such findings were not consistently observed in all trials,^21^ leaving the role of RDN in glucose metabolism controversial.

In preclinical randomized sham-controlled studies, application of this catheter system in diabetic animal models demonstrated that, compared with sham, RDN significantly reduced fasting blood glucose and improved insulin resistance three months post-procedure.^22^ Western blot analyses of hepatic tissue further showed downregulation of key gluconeogenic enzymes (PEPCK and G6Pase) and upregulation of phosphorylated insulin receptor (InsR) and AKT, indicating improved insulin signaling and reduced gluconeogenesis/glycogenolysis.

A recent clinical study confirmed that a six-electrode basket-shaped RF catheter designed by Shanghai Golden Leaf Med Tech (Brattea) Co., Ltd., when applied in the celiac artery, safely and effectively alleviated cancer-related pain.^23^ This non-opioid analgesic approach provided new evidence supporting the safety and feasibility of endovascular sympathetic denervation (EDN). Building on this, an exploratory clinical trial was conducted in 11 patients with T2DM (HbA1c >7.5%) using the same EDN system in the celiac artery (between the celiac trunk ostium and superior mesenteric artery).^24^ EDN achieved a 100% procedural success rate with no device- or procedure-related SAEs or major complications. At 6 months, HbA1c and HOMA-IR were significantly reduced from baseline (9.9% vs 8.0%, P=0.005; 13.3 vs 6.0, P=0.016). Fasting and postprandial glucose also decreased markedly (227.2 vs 181.8 mg/dL, P<0.001; 322.2 vs 205.2 mg/dL, P=0.001). C-peptide release testing indicated improved β-cell function (AUC 0.23 vs 0.28 pmol/mL, P=0.046). Reduced insulin use and improved liver function were also observed. These results suggest that EDN in the celiac artery may safely and effectively improve glycemic control and insulin resistance in T2DM.

Anatomically, abundant sympathetic ganglia exist around the celiac trunk, including fibers regulating pancreatic endocrine secretion. In a canine model of diet-induced diabetes, surgical hepatic sympathetic denervation improved glucose tolerance by up to 60% and increased insulinogenic index by 80% compared to sham.^25^ Hyperinsulinemic-euglycemic clamp studies confirmed improved hepatic glucose metabolism, and benefits persisted at 3 months despite continued high-fat/high-fructose feeding. Hepatic and pancreatic norepinephrine levels decreased significantly, confirming reduced sympathetic tone.^26^ Importantly, counter-regulatory responses to hypoglycemia were preserved, with no adverse changes in lactate, glycerol, free fatty acids, ketone bodies, blood pressure, or heart rate. Thus, hepatic denervation appears to be a safe and effective novel interventional therapy for T2DM and metabolic syndrome.

Currently, EDN targeting the celiac trunk and hepatic artery for T2DM remains at an exploratory stage. Internationally, apart from renal-focused RDN devices, no RF catheter systems are specifically designed for celiac/hepatic arteries. Shanghai Golden Leaf Med Tech (Brattea) Co., Ltd. has developed a next-generation endovascular RF denervation system (generator + catheter), building on its renal platform and pioneering the field in China.

This clinical trial will employ this novel EDN system in T2DM patients with inadequate glycemic control (HbA1c 7.5–10.5%) despite metformin plus 1–3 OADs and/or insulin, to evaluate its safety and efficacy in performing EDN in the celiac trunk and hepatic artery.

## 3.3 Introduction of the Investigational Device

### 3.3.1 Device Characteristics

The Endovascular Denervation System, developed by Shanghai Golden Leaf Med Tech (Brattea) Co., Ltd., consists of the generator and the RF ablation catheter.

The catheter is a basket-shaped, multi-electrode design optimized for variable vessel diameters (no pre-measurement required) with excellent wall apposition, adjustable via handle control. It does not obstruct local blood flow. Six electrodes are arranged in a natural spiral, allowing simultaneous positioning and RF energy delivery with consistent inter-electrode spacing. In combination with the dedicated generator, the catheter delivers RF energy to the vessel wall, generating controlled thermal injury to periarterial sympathetic nerves.

The system provides real-time monitoring of temperature, power, and impedance, with automatic termination if preset thresholds are exceeded, ensuring safety. Ablation data are recorded and stored in real time.

The catheter is manufactured in compliance with national, international, and industry standards, with stable production quality verified by National Medical Products Administration (NMPA) registration testing. Patient-contacting components are made of medical-grade, biocompatible polymers; electrodes are made of precious metals; wiring consists of copper or alloys; structural parts include stainless steel and nitinol. The handle controls electrode expansion to ensure spiral geometry and optimal apposition. All electrodes measure temperature and impedance while delivering RF energy.

The generator features precise impedance measurement, sensitive temperature monitoring, and a user-friendly interface. Parameters are clearly displayed, and automatic shutdown occurs if preset limits are exceeded, ensuring the safety and controllability of the procedure.

### 3.3.2 Structure, Working Principle, and Mechanism of Action of the Endovascular Denervation System

#### 3.3.2.1 Structural Components

**System Composition**

The EDN system consists of the ablation generator (main unit), catheter connection cable, surface electrode connection cable, power cable, foot switch, optional mobile cart, fuses, and user manual (Figure 3-1).

**Figure 3-1. Structural schematic of the EDN generator**

Front (touch display), rear, and side views of the ablation generator.

Front panel components: 1 – RF operation indicator; 2 – alarm indicator; 3 – RF activation button.

Rear/side panel components: A – power switch; B – main power input socket; C – power fuse; D – grounding terminal; E – circuit fuse; G – RS232 serial port; H – Ethernet port; J – USB foot pedal port; K – handle; L – cooling fan; M – SD card slot; N – surface electrode interface; O – expansion interface; P – catheter cable interface

**Catheter Composition**

The EDN ablation catheter mainly comprises a radiopaque distal tip with central pull wire, connector, ablation electrodes, mesh basket frame, protective sheath, seven-lumen shaft, handle housing, handle slider, and connector interface (Figure 3-2).

- The catheter is compatible with an 8F guiding catheter.
- The radiopaque distal tip allows positioning under fluoroscopy.
- The distal basket segment contains six helically arranged ablation electrodes mounted on a mesh frame, all radiopaque. Expansion and retraction are controlled via the handle slider actuating the central pull wire.
- The working segment (distance between electrode 1 and electrode 6) varies according to vessel diameter.
- Each electrode contains internal wiring and a thermocouple sensor, enabling RF energy delivery and simultaneous monitoring of electrode temperature and impedance.
- The handle connector interfaces with the generator via a dedicated catheter connection cable (Figure 3-3)


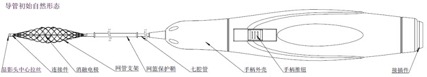


**Figure 3-2. Structural diagram of the EDN catheter**

**Accessories Required**

To complete the EDN procedure, ancillary devices include appropriately sized guidewires, guiding catheters, hemostatic valves, syringes, three-way stopcocks, heparinized saline, and contrast medium (Figure 3-3).


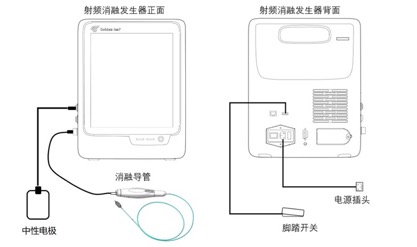


**Figure 3-3. Connection schematic of the catheter, generator, and accessories**

#### 3.3.2.2 Working Principle

**Endovascular Denervation Generator**

(1) Block Diagram and Description

The generator primarily consists of a microcontroller, signal source circuit, power drive circuit, power output circuit, and detection circuits (including those for output power and target temperature), as well as a display and operation control module, as shown in Figure 3-4. It is a high-frequency surgical device that, when connected to the dedicated catheter, delivers radiofrequency (RF) energy, monitors impedance and temperature, and allows parameter settings to control energy output.


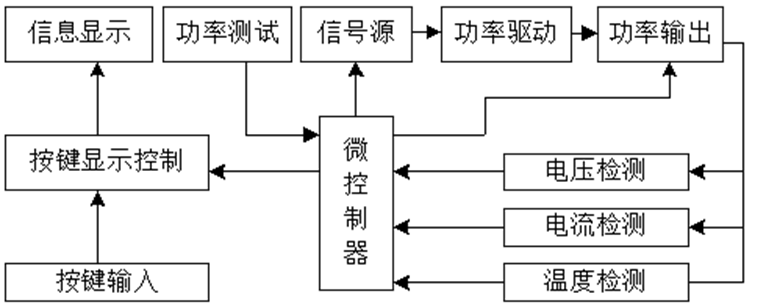


**Figure 3-4. Block diagram of the EDN generator**

(2) RF Generator Display Interface

The display interface is divided into five sections: the self-check interface, settings interface, operation interface, data recording interface, and curve display interface, as shown in Figures 3-5 to 3-9.

1. Self-Check Interface (Figure 3-5)


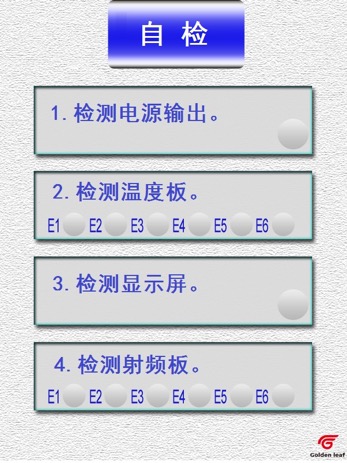


**Figure 3-5 Self-Check Interface**

This interface requires that the SD card be properly installed. Upon power-on, the device automatically performs a self-check of internal modules, including the power supply module, temperature board, LCD screen, and RF board. A check mark indicates that the module has passed the self-check. A cross mark indicates failure. This provides an indication of whether the device can function normally. If a module fails, a fault message will appear (detailed descriptions of error codes can be found in the user manual).

1. Settings Interface (Figure 3-6)

The settings interface allows adjustment of temperature, RF duration, maximum impedance, minimum impedance, and parameter storage. The left and right triangular buttons decrease or increase values, respectively. The save icon button stores the adjusted parameters, which are simultaneously displayed in a reference table. Saved parameters can be recalled by directly selecting the corresponding entry.


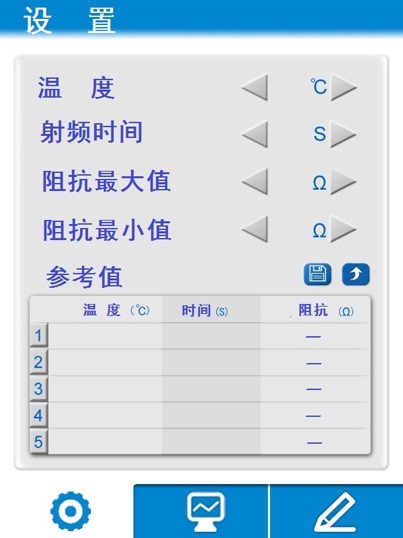


**Figure 3-6 Settings Interface**

Settings include:

① Temperature: Default 40 °C. Pressing the right button increases by 1 °C (long press accelerates), while pressing the left button decreases by 1 °C (long press accelerates). The setting can be adjusted as required for the procedure.

② RF Duration: Default 60 s. Pressing the right button increases by 5 s (long press accelerates), while pressing the left button decreases by 5 s (long press accelerates). Adjust as clinically required.

③ Maximum Impedance: Default 300 Ω. Pressing the right button increases by 10 Ω (long press accelerates), while pressing the left button decreases by 10 Ω (long press accelerates). The protection limit can be adjusted up to a maximum of 300 Ω.

④ Minimum Impedance: Default 100 Ω. Pressing the right button increases by 10 Ω (long press accelerates), while pressing the left button decreases by 10 Ω (long press accelerates). The protection limit can be adjusted down to a minimum of 100 Ω.

⑤ Save Button: Saves the preset values, which will then appear in the reference table. Saved values can be applied by selecting the corresponding entry.

This page can be switched directly to the operation interface or the data recording interface.

1. Operation Interface (Figure 3-7)


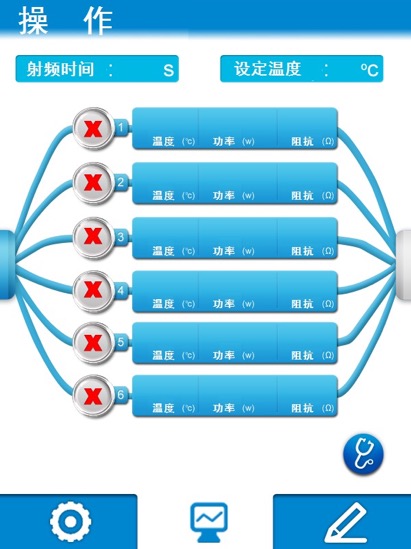


**Figure 3-7 Operation Interface**

The operation interface displays the real-time RF duration and the preset target temperature. In the center of the screen, there are six control buttons corresponding to the six electrodes. When a button is pressed and marked with a check symbol, the corresponding electrode channel is activated. When marked with a cross, the electrode is deactivated. At the lower right corner is the diagnostic button.

Diagnostic Function: During the procedure, individual electrode channels can be selected as needed. Pressing the diagnostic button initiates a gradual power output of 0–0.5 W. The target temperature is observed: A gradual increase indicates good wall contact. No temperature rise, abrupt fluctuations, or impedance exceeding the normal range indicate poor wall contact. In such cases, catheter positioning should be adjusted and the diagnostic test repeated. Any electrode can be selectively disabled. This function is designed to verify proper electrode contact before initiating formal RF ablation.

RF Activation: When the RF switch or foot pedal is pressed for 3 seconds, the system emits an audible signal and the countdown timer begins. Pressing the RF switch or foot pedal again terminates RF output. This interface allows direct switching to the settings interface or the data recording interface.

4) Data Recording Interface (Figure 3-8)


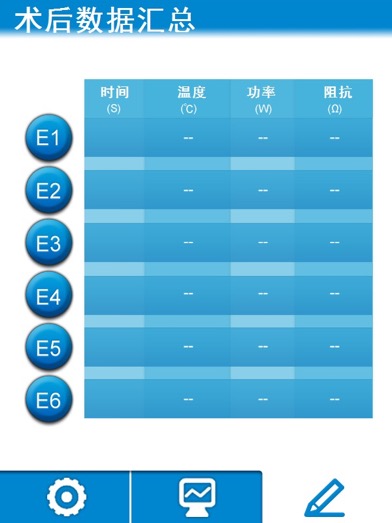


**Figure 3-8 Data Recording Interface**

This interface displays intraoperative data, including RF ablation time, temperature range, power range, and impedance range. Buttons labeled E1–E6 are used to display time-dependent curves of temperature, power, and impedance for each individual electrode during the ablation process. The interface allows direct switching to either the settings interface or the operation interface.

5) Curve Display Interface (Figure 3-9)


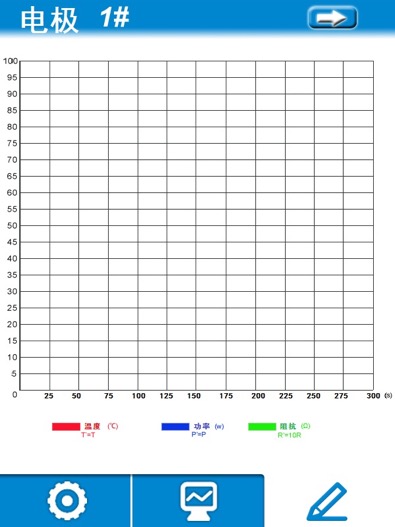


**Figure 3-9 Curve Display Interface**

This interface primarily shows time-dependent curves of temperature, power, and impedance. A “Back” button at the top allows return to the data recording interface. The interface can also be switched directly to the settings interface or the operation interface.

(3) RF Generator Procedural Steps

1) Pre-procedural

① Position the generator and catheter connection cable near the operating table for convenient observation and operation.

② Connect the grounding terminal on the rear panel of the generator to earth using the grounding cable.

③ Connect the prepared catheter and surface electrode pad to the generator.

④ Insert the power cable into the generator’s power socket and connect the other end to the mains outlet.

1. Intra-procedural

① Switch on the generator. The LCD screen will light up and the system will initiate a self-check. After approximately 20 seconds, the device enters standby mode once the self-check is complete.

② Place the prepared surface electrode pad on the patient’s buttock with the conductive side facing upward.

③ Introduce the ablation catheter, confirm correct placement, and ensure that the RF electrodes are in proper contact with the target tissue.

④ Set the required temperature, impedance, and RF duration using the control buttons. The selected values will appear in the corresponding display windows, and successful input will be indicated on the LCD screen.

⑤ Press the diagnostic icon to initiate wall-contact testing. Observe the target temperature: a gradual rise indicates good wall contact, whereas no increase or abrupt fluctuations indicate poor contact. Catheter adjustments may be performed and the test repeated. Any electrode can be selectively disabled by toggling its icon.

⑥ Press and hold the RF button or the foot switch for 3 seconds to start RF ablation. The generator will emit an audible signal and the countdown timer will begin. RF ablation ends automatically when the countdown reaches zero. During ablation, continuously monitor the real-time values of power, impedance, and temperature. If necessary, ablation parameters may be readjusted. To terminate RF prematurely, press the RF button or foot switch again; RF output and the audible signal will stop. To resume RF delivery, restart the activation sequence.

1. Post-procedure

After ablation is completed, first stop RF output. Then disconnect the catheter from the generator, remove the surface electrode pad from the patient, and finally switch off the generator.

**Endovascular Denervation Catheter**

The EDN catheter works in conjunction with the ablation generator to form a closed circuit between the vessel-wall electrodes and the return electrode. RF energy is delivered to perivascular sympathetic nerve fibers, resulting in partial or complete denervation. This reduces sympathetic activity, improves insulin resistance in patients with T2DM, and thereby contributes to improved glycemic control.

The catheter incorporates six helically arranged electrodes, with a working segment diameter ranging from 3 mm to the maximum expansion diameter (see model specifications). The temperature monitoring range is 0–99 °C, with an accuracy of ±3 °C.

Instructions for Use:

① Confirm vascular anatomy and dimensions by arterial angiography, and select an appropriately sized EDN catheter for ablation.

② Using aseptic technique, open the catheter packaging and remove the device. Retract the protective sheath to the seven-lumen shaft, then advance the handle slider to check basket integrity and verify expansion/retraction functionality.

Cautions:

- Do not advance the handle slider to release the basket unless the protective sheath has been fully retracted to the seven-lumen segment.
- Operate the handle slider gently; avoid excessive force.
- If the catheter is damaged, replace it immediately.

③ Immerse the basket segment in heparinized saline. After retracting the basket using the handle slider, reposition the protective sheath to cover the basket and align it with the distal tip.

④ Insert the catheter into the guiding sheath via femoral artery puncture.

⑤ Connect the catheter handle interface to the RF generator using the catheter connection cable.

Cautions:

- If the catheter connection cable has not been sterilized, cover it intraoperatively with a sterile protective sleeve.
- If the “catheter not connected” icon remains on the upper-right corner of the display, disconnect and reconnect the catheter.

⑥ Under fluoroscopic guidance, advance the catheter until the working segment is positioned in the target artery.

⑦ Slowly advance the handle slider to deploy the basket so that the electrodes make contact with the vessel wall. Activate the diagnostic function on the generator to verify wall contact, combined with imaging confirmation.

⑧ Set ablation parameters according to the generator operation manual and initiate RF ablation.

Caution: Once the distal basket is deployed, do not reposition, drag, or rotate the catheter more than 180°. If repositioning is required, fully retract the basket first by advancing the handle slider.

⑨ If ablation is required at other sites, retract the basket completely, reposition the working segment, then repeat wall-contact verification and RF ablation.

⑩ After completing ablation, retract the basket fully by advancing the handle slider. Carefully withdraw the catheter. Perform angiography of the treated artery to exclude complications, then remove the entire catheter system.

⑪ Achieve hemostasis and wound closure at the puncture site following standard interventional protocols.

⑫ After use, dispose of the catheter as medical waste in accordance with local regulations and institutional environmental requirements.

#### 3.3.2.3 Endovascular Denervation System

Endovascular Denervation Generator:

Model: GL-06E (6 × 15 W, temperature control, 12.1-inch resistive LCD touchscreen)

Input Power: 500 VA

Rated Output Power: 6 channels × 15 W

Load Impedance Range: 100 Ω – 300 Ω

Temperature Control Range: 40 °C – 99 °C

Temperature Measurement Range: 0 °C – 99 °C

Maximum Duration of Single RF Ablation: 300 seconds

High-Frequency Leakage Current: The applied part is electrically isolated from ground under both high-frequency and low-frequency conditions. The leakage current flowing from each electrode through a 200 Ω non-inductive resistor to ground must not exceed 150 mA.

Endovascular Denervation Catheter:

Model: GL-6W

(Detailed catheter description to follow.)

| **Model** | **Specification** | **Number of Electrodes** | **Stent Type** | **Length (mm)** | **Maximum Expansion Diameter (mm)** |
| --- | --- | --- | --- | --- | --- |
| GL-6W | 08 | 6 | Net | 1150±10% | 8 |
|  | 12 | 6 | Net | 1150±10% | 12 |

Design: Six electrodes arranged in a helical configuration, with a working segment diameter ranging from 3 mm to the maximum expansion diameter (as specified in the model table).

Temperature Monitoring Range: 0–99 °C

Measurement Accuracy: ±3 °C

#### 3.3.2.4 Packaging and Storage

Endovascular Denervation Generator:

Operating temperature range: –40 °C to +70 °C

Relative humidity range: 10% to 85%

Atmospheric pressure range: 500 hPa to 1060 hPa

Endovascular Denervation Catheter:

Operating temperature range: –30 °C to +60 °C

The product shall be stored in a clean, dry, cool, and well-ventilated environment free from corrosive gases.

#### 3.3.2.5 Shelf Life

The shelf life of the device is specified on the product label. Devices must not be used beyond their indicated shelf life.

#### 3.3.2.6 Mechanism of Action

The EDN catheter operates in conjunction with the ablation generator. By forming a closed circuit between the electrodes in contact with the vessel wall and the return electrode, RF energy is delivered to perivascular sympathetic nerve fibers, causing thermal injury and denaturation of the nerve tissue. This results in partial or complete interruption of sympathetic signaling, reduction of sympathetic activity, improvement in insulin resistance, and ultimately better glycemic control.

The EDN system is equipped with impedance and temperature monitoring functions, ensuring the ablation process remains controlled and safe.

## 3.4 Scope of Application and Related Information

### 3.4.1 Trial Scope

This clinical study will evaluate the safety and efficacy of the endovascular denervation (EDN) system provided by Shanghai Brattea Medical Technology Co., Ltd. for the treatment of T2DM in mainland China.

### 3.4.2 Indications

The EDN system is intended for performing endovascular denervation procedures. The generator, in combination with the dedicated catheter, enables the electrodes to be mechanically apposed to the vessel wall and deliver RF energy to the vessel and perivascular tissues. This creates a continuous energy field that ablates perivascular sympathetic nerves.

Indication: Type 2 Diabetes Mellitus (T2DM).

### 3.4.3 Contraindications

(1) Patients with arterial anatomy unsuitable for ablation.

(2) Patients with allergy to contrast agents.

(3) Patients with abdominal aortic aneurysm.

(4) Patients with significant coagulopathy.

(5) Patients with acute or severe systemic infection.

(6) Pregnant or planning pregnancy.

(7) Patients with severe autonomic neuropathy (e.g., orthostatic hypotension, gastroparesis).

(8) Any condition considered unsuitable for treatment with this device by the investigator (including comorbidities or vascular conditions).

### 3.4.4 Precautions

**Precautions for the RF Generator**

1. Settings
2. Impedance Setting: Upper and lower impedance thresholds (100–300 Ω) can be set to automatically terminate RF delivery if exceeded.
3. Temperature Setting: Target temperature can be set between 40–99 °C.
4. RF Duration Setting: Maximum ablation duration per application is 300 seconds.
5. Control Mode Selection: When temperature-controlled catheters are used, the generator operates in temperature-control mode. The output power is automatically adjusted to maintain the target temperature without exceeding the preset power limit.
6. Audible and Visual Alerts:
7. RF delivery: Audible signal and countdown timer are displayed.
8. Impedance out of range: RF delivery stops, alarm sounds, and impedance window turns red.
9. Termination: RF automatically terminates when preset time is reached or abnormal conditions occur.

① Impedance exceeds preset upper limit: the impedance display window shows “——”; if impedance falls below the preset lower limit, the display background turns red and the corresponding RF channel is automatically switched off.

② Time limit reached: when RF delivery stops upon reaching the preset duration, the buzzer emits an alert sound and the timer resets to the preset value.

③ Target temperature out of measurement range: when the detected temperature exceeds the measurement range, the temperature display window background turns red, the corresponding RF channel is switched off, and temperature, power, and impedance values are all cleared.

④ Output power exceeds maximum allowable value: when power output surpasses the maximum limit, the power display window background turns red, the corresponding RF channel is switched off, and temperature, power, and impedance values are all cleared.

1. Fault: In case of power supply or system failure, the LCD display shows red background alarms, and the system halts output.
2. Display Functions: The LCD displays preset and measured values for impedance, power, temperature, and time, as well as cumulative RF duration and cycle counts, with error and termination messages.
3. Data Storage: An SD card is used for storing preset and saved parameters (impedance, power, temperature, time, and control mode).
4. Communication Port: One RS-232 interface is available, compliant with EN 60601-1-1, for software upgrades and diagnostic purposes.

**Precautions for the RF Catheter**

(1) The catheter is to be used exclusively with the dedicated EDN generator.

(2) It is restricted to use by physicians trained in angiography and interventional techniques, and trained in this product.

(3) Do not use expired products.

(4) The catheter is a sterile, single-use device. Do not reuse or resterilize.

(5) The catheter is sterilized with ethylene oxide. Do not use if sterile packaging is damaged.

(6) The system must be used in conjunction with high-quality DSA imaging equipment.

(7) Do not release the basket by pushing the handle slider unless the protective sheath has been retracted.

(8) Dispose of used catheters and packaging as medical waste according to local regulations.

(9) Rated Voltage: 135 Vp

# Study Objective

The purpose of this clinical trial is to evaluate the safety and efficacy of EDN performed in the celiac trunk and hepatic artery using the EDN generator and catheter developed and manufactured by Shanghai Brattea Medical Technology Co., Ltd. for the treatment of T2DM.

# Study Design

## 5.1 Overall Design and Rationale

The feasibility clinical trial of endovascular denervation for the treatment of T2DM is a prospective, multicenter, single-arm feasibility study. The study will employ the EDN system (including generator and catheter) developed by Shanghai Brattea Medical Technology Co., Ltd. to perform EDN in the celiac trunk and hepatic artery for T2DM treatment.

As this is an early feasibility study of EDN for T2DM, and no device with the same indication has yet been approved for registration in China, a single-arm design was selected. Additionally, the primary study endpoints are not significantly influenced by placebo effects or psychological factors, making this design appropriate.

During EDN, anticoagulation will be administered according to clinical standards. Antiplatelet therapy will be used pre- and post-procedure to prevent thrombosis. Dynamic safety monitoring will be conducted throughout the trial to minimize patient risk. All enrolled subjects who undergo EDN will be followed for 24 months post-procedure.

## 5.2 Subject Selection

A total of 30 patients with T2DM who have inadequate glycemic control despite antidiabetic medications and are eligible for EDN treatment will be enrolled in this feasibility study. Clinical inclusion criteria and clinical/angiographic exclusion criteria are described in Sections 5.2.1 and 5.2.2, respectively.

Only subjects who meet all clinical inclusion criteria and none of the exclusion criteria will be considered for enrollment in this study.

### 5.2.1 Inclusion Criteria

Subjects may be enrolled only if they meet all of the clinical inclusion criteria (see Table 5-1) and none of the exclusion criteria (see Section 5.2.2).

**Table 5-1 Inclusion Criteria**

| **Clinical Inclusion Criteria** | 1. Age between 18 and 65 years (inclusive). 2. The subject understands the requirements of this clinical trial and the treatment, agrees to and is able to complete all required follow-up assessments, and has signed the informed consent form prior to any trial-related procedures or interventions. 3. Diagnosed with T2DM for 1–15 years (according to WHO diagnostic criteria). 4. On stable treatment for ≥3 months with metformin (≥1000 mg/day) in combination with 1–3 oral antidiabetic drugs (OADs) and/or insulin (any dose). Eligible OADs include: insulin secretagogues (sulfonylureas/meglitinides), thiazolidinediones (TZDs), and α-glucosidase inhibitors (see note). The combined OAD dose must be at least half of the maximum approved dose stated in the drug label. 5. Despite ≥3 months of OAD therapy, glycemic control remains inadequate, with HbA1c between 7.5% and 10.5% (as determined at baseline). 6. Body mass index (BMI) between 18 and 40 kg/m² (inclusive).   Note: Subjects receiving α-glucosidase inhibitors must undergo a 2-week washout period before enrollment. |
| --- | --- |

###

### 5.2.2 Exclusion Criteria

Subjects meeting any of the exclusion criteria listed in Table 5-2 will be excluded from this clinical study.

**Table 5-2 Exclusion Criteria**

| **Clinical Exclusion Criteria** | I. Subjects with any of the following medical history or conditions:  1.Type 1 diabetes mellitus, latent autoimmune diabetes in adults (LADA), or any form of secondary diabetes.  2.History of aortic disease (e.g., aneurysm or dissection) or aortic surgery (including prior celiac denervation).  3.Baseline CTA showing aortic aneurysm/dissection, abnormal hepatic artery anatomy, or other vascular abnormalities unsuitable for ablation (e.g., severe tortuosity or stenosis, intraluminal thrombus, unstable plaques, as judged by the investigator).  4.≥2 self-reported or documented episodes of severe hypoglycemia within the past 6 months (defined as hypoglycemia with severe cognitive impairment requiring assistance).  5.≥1 documented episode of hyperglycemic crisis within the past 6 months requiring hospitalization (including diabetic ketoacidosis or hyperosmolar coma).  6.Severe diabetic complications considered unsuitable for enrollment by the investigator (e.g., advanced retinopathy, nephropathy, vasculopathy, neuropathy, or diabetic foot).  7.Major adverse cardiovascular or cerebrovascular event (MACCE) within the past 6 months, including cerebrovascular accident (CVA), transient ischemic attack (TIA), heart failure (NYHA class III–IV), acute myocardial infarction, or unstable angina requiring hospitalization (including history of CABG or coronary stent implantation), as well as uncontrolled or severe arrhythmias.  8.Severe autonomic neuropathy (e.g., orthostatic hypotension, gastroparesis).  9.Uncontrolled severe hypertension (SBP ≥160 mmHg or DBP ≥100 mmHg) or hypotension (BP <90/50 mmHg).  10.Renal insufficiency or history of renal failure with baseline estimated glomerular filtration rate (eGFR) <60 mL/min/1.73 m².  11.Chronic active hepatitis, severe hepatobiliary disease (including cirrhosis), or hepatic insufficiency (ALT and/or AST >3× ULN, or total bilirubin >2× ULN).  12.Acute or chronic pancreatitis during screening or baseline period.  13.Acute systemic infection during screening or baseline period.  14.History of bleeding tendency or coagulopathy (PT, APTT, or INR >2× ULN; platelet count <80×10⁹/L or ≥700×10⁹/L).  15.Active peptic ulcer disease or gastrointestinal bleeding within the past 3 months.  16.Symptomatic cholelithiasis (including gallbladder, extrahepatic bile duct, or intrahepatic bile duct stones) without effective treatment (e.g., cholecystectomy, bile duct stone removal, internal/external drainage).  17.Other endocrine or metabolic disorders, including hyperthyroidism, hypothyroidism, acromegaly, or Cushing’s syndrome.  18.Autoimmune disease.  19.Active or high-risk malignancy, or life expectancy <12 months.  20.Major surgery within the past 3 months.  21.Hemoglobinopathies or hemolytic anemia that may interfere with evaluation of primary endpoints.  22.Psychiatric disorders that preclude compliance with study procedures.  Note: Subjects not meeting inclusion criteria during screening may be rescreened if deemed appropriate by the investigator.  II. Subjects with prior or expected use of the following medications or treatments: 23. GLP-1 receptor agonists, DPP-4 inhibitors, or SGLT-2 inhibitors within 3 months prior to screening. 24. Systemic or intra-articular corticosteroid therapy within 3 months prior to screening (excluding topical, inhaled, or ophthalmic use). 25. Use of weight-loss drugs (e.g., orlistat) or other weight-reducing interventions (e.g., herbal medications, acupuncture, diet therapies) within 3 months prior to screening. 26. History or expectation of undergoing bariatric surgery (e.g., gastrectomy, liposuction). 27. Use of centrally acting sympatholytics or anticonvulsants within 3 months prior to screening or expected during the study. 28. Subjects requiring long-term anticoagulation who cannot undergo perioperative heparin bridging.  III. Subjects with the following conditions: 29. Body weight fluctuation >10% within the past 3 months. 30. Allergy or contraindication to contrast agents that cannot be adequately managed with premedication. 31. Known allergy to the investigational device or trial-related medications. 32. Participation in another clinical study within 3 months prior to enrollment. 33. Planned participation in another drug or device clinical trial within 24 months post-baseline procedure. 34. Pregnant or lactating women, or women planning pregnancy within 2 years (all women of childbearing potential must undergo a pregnancy test within 7 days prior to the baseline procedure). 35. Anticipated major changes in diet or exercise habits during the study period (e.g., due to religious practices, occupational reasons, or weight-loss programs). 36. Irregular day-night schedule (e.g., shift workers). 37. History of alcohol abuse, drug abuse, or substance dependence. 38. Severe blood loss or planned regular transfusion of blood products within 3 months prior to enrollment or during the study. 39. Any other condition judged by the investigator to pose risk to subject safety or significantly interfere with study outcomes. |
| --- | --- |
| **Angiographic Exclusion Criteria** | 1. Presence of aortic aneurysm or dissection confirmed by angiography prior to EDN.   41. Vascular anatomy or condition considered unsuitable for ablation by the investigator (e.g., severe tortuosity or stenosis, abnormal anatomy, intraluminal thrombus, unstable plaques). |

### 5.2.3 Subject Withdrawal Criteria and Procedures

#### **5.2.3.1 Enrollment Time Point**

A subject is considered eligible for the study after signing an informed consent form approved by the ethics committee, meeting all inclusion criteria, and not meeting any exclusion criteria. The official enrollment time point is defined as the moment the EDN catheter is introduced into the subject’s body.

#### **5.2.3.2 Withdrawal**

Although withdrawal without cause is discouraged, subjects may withdraw from the clinical trial at any time, with or without explanation, and this will not affect their future medical care.

The investigator may also discontinue or withdraw a subject from the study for the following reasons:

Occurrence of an unanticipated adverse device effect (UADE) that poses significant or unreasonable risk to the subject.

- Any condition arising during the trial that could harm the subject (e.g., pregnancy, inability to continue follow-up assessments, hypersensitivity to the study device or contrast medium).

Inability of the subject to complete the required study procedures or follow-up.

- All subjects enrolled in the trial (including those who withdraw or are lost to follow-up) will be documented. Withdrawn subjects will not be replaced, and no additional follow-up will be conducted. Investigators should document the reason for withdrawal whenever possible.
- If withdrawal is related to the safety or efficacy of the study device or procedure, the investigator should seek subject consent to continue clinical monitoring outside the trial. Data collected prior to withdrawal will be retained and included in analyses, but no further data will be collected after withdrawal.

#### **5.2.3.3 Enrollment Control**

The study will enroll at least 30 subjects, with no restrictions on the number of subjects recruited at each participating center.

## 5.3 Evaluation Methods

### 5.3.1 Primary Endpoints

#### **5.3.1.1 Primary Safety Endpoint**

The primary safety endpoint is the incidence of composite major adverse events (MAE)* related to the investigational device and/or ablation procedure occurring intraoperatively or within 30 days post-baseline procedure.

- MAE is defined to include the following events:
- Hypoglycemia or hyperglycemia requiring hospitalization.
- Vascular complications requiring surgical repair, interventional treatment, thrombin injection, or blood transfusion.
- Celiac/hepatic artery dissection or perforation requiring intervention.
- Significant embolic events leading to end-organ damage or requiring intervention to prevent end-organ damage.
- Acute liver injury or liver failure.
- All-cause mortality.

#### **5.3.1.2 Primary Efficacy Endpoint**

The primary efficacy endpoint is the change in glycated hemoglobin (HbA1c) from baseline to 6 months post-procedure.

### 5.3.2 Secondary Endpoints

The following will be assessed at 1, 3, 6, 12, and 24 months post-procedure compared with baseline:

1. Changes in:

- HbA1c (at 1, 3, 12, and 24 months).
- β-cell function and insulin resistance (HOMA-β and HOMA-IR; formulas provided in the data collection table).
- Mean glucose, postprandial glucose excursions, nocturnal glucose excursions, and time in range (TIR) measured by 14-day continuous glucose monitoring (CGM).
- Lipid profile (triglycerides, total cholesterol, HDL-C, LDL-C).
- Liver function tests (ALT, AST, ALP, GGT).
- Renal function tests (BUN, serum creatinine, eGFR).
- Oral glucose tolerance test (OGTT) and insulin/C-peptide secretion tests (five-point sampling at 0, 30, 60, 90, and 120 minutes), assessed at 6, 12, and 24 months.
- Proportion of subjects achieving HbA1c <7.0% and ≤6.5%.
- Proportion of subjects achieving HbA1c <7.0% without hypoglycemia/severe hypoglycemia or weight gain (composite endpoint).
- Changes in type/dose of antidiabetic medications and proportion of subjects with medication reduction.
- Incidence of hypoglycemia and severe hypoglycemia (blood glucose <3.9 mmol/L).
- Incidence of new-onset significant stenosis (>50%) in the celiac artery, hepatic artery, or branches at 6 months post-procedure, confirmed by digital subtraction angiography (DSA) if CTA indicates abnormalities.
- Incidence of adverse events (AEs) and serious adverse events (SAEs) related to the investigational device and/or procedure at baseline, 7 days (or discharge), 1, 3, 6, 12, and 24 months.
- Incidence of major adverse cardiovascular and cerebrovascular events (3MACE: cardiovascular death, myocardial infarction, ischemic stroke) at 12 and 24 months.
- Changes in quality of life/lifestyle assessment using the EQ-5D-5L questionnaire at baseline and 6 months post-procedure.

### 5.3.3 Additional Assessments

In addition to the above endpoints, the following parameters will be collected at baseline and during follow-up visits (as detailed in the clinical data collection table) and analyzed for changes:

- Body weight and BMI.
- Waist circumference and waist-to-hip ratio.
- Office blood pressure.
- Changes in heart rate variability (24-hour Holter monitoring).
- Plasma catecholamine levels (including epinephrine and norepinephrine).
- Device performance evaluation (as assessed by the operator).

## 5.4 Study Flow

### 5.4.1 Study Flowchart

This feasibility clinical study of endovascular denervation for T2DM will recruit up to 30 subjects across a maximum of three centers in mainland China.

**Enrollment period:** approximately 3 months.

**Follow-up period:** 24 months.

All subjects will be screened according to inclusion and exclusion criteria and enrolled in a non-randomized manner.

Clinical follow-up visits will be conducted at the following time points:

Day 7 post-procedure or before discharge (whichever comes first), 30 days (±7 days), 3 months (±14 days), 6 months (±30 days), 12 months (±30 days), and 24 months (±30 days) post-procedure.

**Screening+Baseline^*^**

**Angiography**

**+**

**EDN**

**N = 10**

**Inform consent**

**Inclusion criteria**

**Washout^*^**

**Baseline Data**

**Follow-ups**

**Enrollment+Procedure**

**7 days**

**3 months**

**12 months**

**24 months**

**6 months**

**30 days**

**Figure 5-1 Study Flowchart**

* Subjects eligible for enrollment who are taking α-glucosidase inhibitors must undergo a 2-week washout period before being considered for enrollment.

Notes: A subject is considered to have completed the study if they have completed the 24-month follow-up, withdrawn (due to death or loss to follow-up), or exceeded the last follow-up window (i.e., >30 days beyond the scheduled visit window). For subjects who withdraw early, data collected up to the point of withdrawal will be retained and analyzed, but no further data will be collected thereafter.

All subjects must discontinue metformin on the day of CTA or DSA examination.

###

### 5.4.2 Data Collection

Please note:

- The time window for the 30-day follow-up is ±7 days.
- The time window for the 3-month follow-up is ±14 days.
- The time windows for the 6-, 12-, and 24-month follow-ups are ±30 days.
- The table below (Table 5-2) summarizes all procedures and assessments required by the study protocol throughout the trial.

**Table 5-2 Clinical Data Collection Schedule**

|  | | **Screening^a^** | **Baseline (≤14 days pre-procedure)** | **Baseline Procedure / Enrollment (Day 0)** | **Follow-ups** | | | | | |
| --- | --- | --- | --- | --- | --- | --- | --- | --- | --- | --- |
|  |  |  |  |  | **Post-op 7 days or discharge** | **Post-op 30 days^b^ (±7)** | **Post-op 3 months^b^ (±14)** | **Post-op 6 months^b^ (±30)** | **Post-op 12 months^b^ (±30)** | **Post-op 24 months^b^ (±30)** |
| **On-site visit** | | X | X | X |  | X | X | X | X | X |
| **Informed consent** | | X |  |  |  |  |  |  |  |  |
| **Demographics (age, sex, race, etc.)** | | X |  |  |  |  |  |  |  |  |
| **Medical history** | | X |  |  |  |  |  |  |  |  |
| **Inclusion / exclusion criteria** | | X | X | X |  |  |  |  |  |  |
| **Physical exam (weight, height, waist & hip circumference, office BP)** | | X |  |  |  | X | X | X | X | X |
| **Antidiabetic medication / insulin use** | | X | X | X | X | X | X | X | X | X |
| **Other treatments (CV meds, anticoagulants, antiplatelets)** | | X | X | X | X | X | X | X | X | X |
| **EQ-5D-5L questionnaire** | |  | X |  |  |  |  | X |  |  |
| **Lab tests** | **CBC** | X |  |  | X | X | X | X | X | X |
|  | **Pregnancy test (if applicable)** |  | X |  |  |  |  |  |  |  |
|  | **Renal function^c^** | X |  |  | X | X | X | X | X | X |
|  | **Coagulation profile** | X |  |  |  |  |  |  |  |  |
|  | **OGTT^d^** |  | X |  |  |  |  | X | X | X |
|  | **14-day CGM^e^** |  | X^q^ |  |  | X | X | X | X | X |
|  | **HbA1c** | X^o^ | X^p^ |  |  | X | X | X | X | X |
|  | **Fasting glucose** |  |  |  |  | X | X |  |  |  |
|  | **Fasting insulin** |  |  |  |  | X | X |  |  |  |
|  | **HOMA-β and HOMA-IR^f^** |  | X |  |  | X | X | X | X | X |
|  | **Lipid profile^g^** |  | X |  |  | X | X | X | X | X |
|  | **Liver function^h^** | X |  |  | X | X | X | X | X | X |
|  | **Catecholamines^i^** |  | X |  |  | X | X | X | X | X |
| **HRV (24-h Holter)^j^** | |  | X |  |  | X |  | X |  |  |
| **Radiographics** | **Celiac CTA^k^** | X |  |  |  |  |  | X |  |  |
|  | **Celiac angiography (DSA)^l^** |  |  | X |  |  |  | X |  |  |
| **EDN procedure/device info^m^** | |  |  | X |  |  |  |  |  |  |
| **AE/ADE/SAE/SADE assessment^n^** | |  |  | X | X | X | X | X | X | X |

Notes and Annotations

Remarks: If a subject does not meet the inclusion criteria during the screening period, re-screening may be conducted after investigator assessment.

Annotations:

a. Subjects taking α-glucosidase inhibitors during screening require a 2-week washout period before enrollment.

b. All follow-up visits are calculated from the date of the baseline procedure.

c. Renal function includes BUN, serum creatinine, and eGFR (calculated using CKD-EPI formula).

d. OGTT: 5-point sampling (0, 30, 60, 90, 120 minutes) with glucose, insulin, and C-peptide measurement; indices include Matsuda Index, insulin clearance, insulinogenic index, C-peptide index, late insulin response, and oral disposition index.

e. 14-day CGM for mean glucose, postprandial excursions, nocturnal excursions, and time-in-range (TIR).

f. β-cell function and insulin resistance assessed using HOMA-β and HOMA-IR. HOMA-β = 20 × FINS / (FBG – 3.5); HOMA-IR = (FBG × FINS) / 22.5. (FINS = fasting insulin μU/mL, FBG = fasting glucose mmol/L).

g. Lipid profile includes TG, TC, HDL-C, LDL-C.

h. Liver function tests include ALT, AST, ALP, TBil, GGT.

i. Catecholamines include norepinephrine and epinephrine.

j. HRV evaluated by 24-hour Holter monitoring.

k. Screening CTA of the celiac trunk excludes vascular abnormalities.

l. Pre-EDN angiography excludes vascular anomalies; at 6 months, DSA required if CTA suggests >50% stenosis; no DSA if CTA is normal.

m. Device and procedural information collected for device performance evaluation.

n. Includes major adverse events (MAE), major adverse cardiovascular and cerebrovascular events (3MACE), and hypo-/hyperglycemia.

o. HbA1c results within 1 month prior to screening are acceptable.

p. HbA1c results within 14 days prior to baseline procedure are acceptable; otherwise, repeat testing required.

q. Baseline CGM within 20 days prior to procedure is acceptable; follow-up CGM initiated at visit.

Laboratory tests in this trial include:

- CBC: WBC, RBC, LY, NEU, HCT, Hb, PLT

- Pregnancy test

- Renal function: BUN, SCr, eGFR

- Coagulation profile: PT, APTT, TT, FIB, INR

- Fasting glucose, fasting insulin, fasting C-peptide

- OGTT

- HbA1c

- Lipid profile: TC, HDL-C, LDL-C, TG

- Liver function: ALP, ALT, AST, TBil, GGT

- Catecholamines: norepinephrine, epinephrine

| **Test Item** | **Screening** | **Baseline (≤14 days pre-procedure)** | **Post-op 7 days or discharge** | **Post-op 1 month (±7 days)** | **Post-op 3 months (±14 days)** | **Post-op 6 months (±30 days)** | **Post-op 12 months (±30 days)** | **Post-op 24 months (±30 days)** |
| --- | --- | --- | --- | --- | --- | --- | --- | --- |
| CBC – 3 ml | X |  | X | X | X | X | X | X |
| HbA1c – 3 ml | X |  |  | X | X | X | X | X |
| Renal + Liver function – 5 ml | X |  | X |  | X | X | X | X |
| Coagulation – 3 ml | X |  |  |  |  |  |  |  |
| Pregnancy test – 5 ml |  | X |  |  |  |  |  |  |
| Lipid profile – 5 ml |  | X |  |  | X | X | X | X |
| OGTT + Insulin + C-peptide – 42.5 ml (3.5 ml for glucose each time point, 5 ml for insulin + C-peptide) |  | X |  |  |  | X | X | X |
| Fasting insulin – 5 ml |  |  |  | X | X |  |  |  |
| Fasting glucose – 3.5 ml |  |  |  | X | X |  |  |  |
| Catecholamines – 5 ml |  | X |  | X | X | X | X | X |

### 5.4.3 Treatment Assignment

After signing the informed consent form (ICF) approved by the ethics committee, and meeting all inclusion criteria while not meeting any clinical exclusion criteria, subjects will be considered for enrollment. Subjects receiving α-glucosidase inhibitors must undergo a 2-week washout period before enrollment.

This clinical trial has no control group. All eligible subjects will undergo baseline angiography during the procedure. If no angiographic exclusion criteria are met, they will receive EDN treatment using the endovascular denervation system developed by Shanghai Brattea Medical Technology Co., Ltd. The enrollment time point is defined as the insertion of the EDN catheter into the guiding sheath.

### 5.4.4 Subject Screening

Before any trial-specific screening procedures, eligible subjects for EDN will receive a detailed explanation of the study from the investigator, and will be required to sign the ICF. After signing, the subject will be assigned a screening number and recorded in the screening log.

If a subject does not meet inclusion criteria during screening, re-screening may be conducted at the investigator’s discretion. Investigators must maintain complete screening records for all subjects, including both those deemed eligible and those who failed screening.

### 5.4.5 Informed Consent

Prior to any study-specific assessments or procedures, subjects who meet the clinical inclusion criteria must sign the ICF approved by the ethics committee.

Investigators must allow sufficient time for subjects to review the ICF and address any questions raised. Even if a subject signs the ICF, study staff must explain that the final eligibility will be determined by angiographic findings, which may exclude the subject from participation.

### 5.4.6 Screening Period

Only after signing the ICF may a subject undergo screening. Screening is intended to confirm whether the subject meets inclusion and exclusion criteria.

The screening process includes review of the subject’s medical history and diagnostic records, as well as collection of the following information (unless otherwise specified):

- Confirmation of compliance with inclusion criteria and absence of exclusion criteria.
- Demographic data: age, sex, ethnicity (per local regulatory requirements).
- Physical examination: height, weight, waist and hip circumference, office blood pressure.
- Medical history: duration of diabetes, complications, comorbidities, surgical history.
- Antidiabetic medication and insulin use.
- Concomitant treatments (e.g., cardiovascular drugs, anticoagulants, antiplatelets).
- Laboratory tests:
- Complete blood count (CBC).
- Coagulation profile.
- Renal function: BUN, serum creatinine, eGFR.
- Liver function: ALT, AST, ALP, total bilirubin, GGT.
- HbA1c.
- Celiac CTA.*

*CTA results within 30 days prior to baseline procedure are acceptable. Metformin must be withheld on the day of CTA.

**Washout Period**

Subjects who meet clinical screening criteria but are taking α-glucosidase inhibitors must discontinue them for 2 weeks prior to enrollment. During this washout period, all other antidiabetic medications must remain unchanged. Subjects requiring a washout must complete this period before entering the baseline phase.

### 5.4.7 Baseline Period (within 14 days pre-procedure)

For subjects who pass screening, the following data must be collected within 14 days prior to the baseline procedure:

- Antidiabetic medication and insulin use.
- Concomitant treatments (cardiovascular, anticoagulant, and antiplatelet therapies).
- Laboratory assessments:
- Glucose metabolism: 5-point OGTT, 14-day CGM (may start up to 16 days before procedure, but duration must be ≥14 days), and HbA1c.
- β-cell function and insulin resistance: 5-point insulin and C-peptide test, HOMA-IR, HOMA-β.
- Lipid profile: triglycerides, total cholesterol, HDL-C, LDL-C.
- Plasma catecholamines: norepinephrine and epinephrine.
- Pregnancy test (serum) for women of childbearing potential within 7 days pre-procedure.
- Heart rate variability: 24-hour Holter monitoring.
- Quality of life/lifestyle assessment: EQ-5D-5L questionnaire.
- Reconfirmation of inclusion and exclusion criteria.

#### 5.4.7.1 Concomitant Medication and Lifestyle Management

From screening until the 24-month follow-up, all antidiabetic medications (OADs and insulin, including dose adjustments, interruptions, or discontinuations) as well as concomitant medications required by the protocol must be recorded in the electronic case report form (eCRF).

Treating physicians may adjust antidiabetic therapy or prescribe other concomitant medications according to standard clinical practice. Subjects will also receive counseling and guidance on lifestyle management for diabetes.

#### 5.4.7.2 Concomitant Antidiabetic Therapy and Lifestyle Management

Subjects must be on stable OAD therapy for at least 3 months prior to the baseline procedure (subjects taking α-glucosidase inhibitors must undergo a 2-week washout before enrollment). After EDN, OADs and insulin regimens will be adjusted by the investigator according to blood glucose levels and guideline recommendations (see Sections 5.4.7.3 and 5.4.7.4). All changes in OADs and insulin type/dose must be documented at follow-up visits.

#### 5.4.7.3 Principles for Rescue Treatment of Hyperglycemia

For subjects meeting any of the following hyperglycemia definitions, and where no definite cause can be identified, investigators may initiate insulin therapy on top of existing treatment as rescue therapy, based on clinical judgment.

**Study Period Criteria (≥2 weeks of 4-point SMBG daily mean):**

- 4 weeks post-EDN: FBG ≥ 13.9 mmol/L
- 8 weeks post-EDN: FBG ≥ 11.1 mmol/L
- 8 weeks post-EDN: HbA1c ≥ 8.5%

**Recommended principles of rescue treatment for hyperglycemia:**

- Initiate insulin therapy in addition to existing treatment, including intensifying basal insulin dose and/or adding short-acting or rapid-acting insulin.
- Adjust basal insulin dose every 3–5 days according to fasting blood glucose (FBG). Increase by 1–4 U per adjustment until target FBG is achieved. The maximum basal insulin dose should not exceed 0.5–0.6 U/kg/day.

#### 5.4.7.4 Principles for Medication Adjustment in Hypoglycemia

Hypoglycemia is an adverse event requiring special attention after EDN. Hypoglycemia is defined as blood glucose <3.9 mmol/L.

**Management principles:**

- If hypoglycemia occurs, immediately administer 15–20 g glucose and recheck blood glucose after 15 minutes.
- In subjects receiving insulin therapy, glycemic targets should be relaxed to strictly avoid recurrent hypoglycemia for several weeks, to partially reverse hypoglycemia unawareness and reduce future risk.
- Medication reduction should follow the sequence: Insulin (if applicable) → Sulfonylureas → Glinides → Thiazolidinediones → Metformin (final adjustment of metformin at investigator’s discretion).

#### 5.4.7.5 Principles of Lifestyle Management

During the study, qualified healthcare professionals will provide diabetes management counseling at each visit, including dietary and exercise guidance, and education on the signs, symptoms, and treatment of hypoglycemia (if it occurs).

**Lifestyle management principles:**

- Subjects should continue their usual physical activity and follow a healthy diet plan (consistent quantity and timing of meals) throughout the study.
- Dietary counseling may be reviewed as needed during the study.
- Apart from lifestyle measures required for diabetes care, subjects should not initiate structured diet and/or exercise weight-loss programs during the study.

#### 5.4.7.6 Periprocedural Medication Requirements

**Analgesia/Sedation for EDN Procedure**

In addition to local anesthesia at the puncture site, appropriate analgesic and sedative medications should be administered during EDN according to local standards to reduce pain/discomfort, maintain hemodynamic stability, and ensure procedural tolerability and efficacy. Oxygen supplementation via mask or nasal cannula is recommended. Morphine sulfate or fentanyl combined with midazolam may be used (dosages determined by investigators per standard care and patient condition). Additional doses may be administered during ablation if necessary.

**Antiplatelet Therapy**

To minimize thrombotic complications, antiplatelet therapy (monotherapy) should be initiated before the procedure, such as aspirin (ASA) or a P2Y12 inhibitor (clopidogrel preferred; ticlopidine may be used if intolerant). Subjects already receiving ≥72 hours of ASA or a P2Y12 inhibitor do not require a loading dose.

Otherwise, a loading dose must be given before or no later than 2 hours after the baseline procedure.

**Recommended minimum loading doses:** ASA 300 mg, clopidogrel 300 mg.

**Anticoagulation**

Standard intraoperative heparinization should be administered, or other anticoagulation selected as clinically appropriate. Intraoperative activated clotting time (ACT) should be maintained at ~250 seconds.

Post-procedure antiplatelet therapy should be continued for at least 1 week post-procedure. Minimum daily maintenance doses: ASA 75 mg/day or clopidogrel 75 mg/day.

Note: To reduce bleeding risk, subjects on long-term anticoagulation should not receive additional antiplatelet therapy.

### 5.4.8 Enrollment and Baseline Procedure

Subjects meeting all inclusion criteria and no clinical exclusion criteria must complete the following before enrollment:

- Angiography of the abdominal aorta and its branches, with documentation of findings.
- Assessment of angiographic exclusion criteria.

Enrollment is confirmed only when no angiographic exclusion criteria are met. The enrollment time point is defined as insertion of the EDN catheter into the subject.

The baseline procedure starts with insertion of the guiding catheter and ends with its removal after final angiography.

The following intraoperative data must be collected and recorded in the eCRF:

- Procedure date and time (start = guiding catheter insertion; end = removal after final angiography).
- Device information: generator model/serial number, catheter model/batch/serial number (see Table 5-3).

**Table 5-3 Device performance evaluation**

| **Generator Evaluation** | |
| --- | --- |
| Mode | GL-06E |
| Generator appearance intact and in good condition | □ Good □ Not good |
| Generator can be switched on/off normally | □ Yes □ No |
| Generator can perform self-check at startup | □ Yes □ No |
| Generator allows normal parameter setting | □ Yes □ No |
| Generator can detect surface electrodes | □ Yes □ No |
| Generator can recognize ablation catheter | □ Yes □ No |
| Generator can perform RF ablation normally | □ Yes □ No |
| Surgical interface can display ablation data in real time | □ Yes □ No |
| Ablation data can be stored normally | □ Yes □ No |
| Generator can terminate RF ablation normally after countdown | □ Yes □ No |
| **Catheter Evaluation** | |
| Catheter package intact | □ Intact □ Damaged |
| Catheter appearance normal | □ Yes □ No |
| Catheter basket expansion/retraction performance normal | □ Yes □ No |
| Catheter can smoothly pass through the sheath | □ Yes □ No |
| Catheter pushability in vessel | □ Good □ Fair □ Poor |
| Catheter flexibility | □ Appropriate □ Slightly soft □ Slightly stiff □ Too soft □ Too stiff |
| Electrode visibility under X-ray | □ Good □ Fair □ Poor |
| Catheter positioning controllability | □ Good □ Fair □ Poor |
| Connection cable matches relevant equipment | □ Yes □ No |
| Catheter can perform RF ablation normally | □ Yes □ No |
| Catheter integrity after withdrawal | □ Complete □ Incomplete (please describe): ______ |

- Vessels treated, ablation sites, number of ablation points, ablation duration, and parameters (temperature, power, impedance).
- Other devices or adjunctive therapies used intraoperatively.
- Intraoperative and postoperative medications, including analgesics/sedatives, antidiabetics/insulin, anticoagulants, antiplatelets, and ACT.
- Adverse event (AE) assessment and management, including AE, SAE, SADE, UADE, USADE, and ADE.
- Reconfirmation of inclusion/exclusion criteria.

Note: Metformin must be withheld on the day of DSA.

### 5.4.9 Post-op Day 7 or Before Discharge

All enrolled subjects must undergo CBC and renal/liver function testing at Day 7 post-procedure or before discharge (whichever comes first). Concomitant use of antidiabetics, insulin, and anticoagulants/antiplatelets must be documented. AE/ADE/SAE/SADE and device deficiencies must also be assessed and recorded.

### 5.4.10 30-Day Follow-up (±7 days)

At 30 days post-procedure, subjects must return for follow-up including:

- Physical exam (height, weight, waist & hip circumference, office BP).
- Antidiabetic medication/insulin use.
- Other treatments (cardiovascular, anticoagulant, antiplatelet therapies).
- Laboratory tests: CBC, renal function (BUN, creatinine, eGFR), fasting glucose & insulin, 14-day CGM, HbA1c, HOMA-IR & HOMA-β, lipid profile, liver function (ALT, AST, ALP, TBil, GGT), plasma catecholamines.
- HRV: 24-hour Holter monitoring.
- AE assessment and management (AE, SAE, SADE, UADE, USADE, ADE).

### 5.4.11 3-Month Follow-up (±14 days)

At 3 months post-procedure, subjects must return for follow-up including:

- Physical exam (height, weight, waist & hip circumference, office BP).
- Antidiabetic medication/insulin use.
- Other treatments (cardiovascular, anticoagulant, antiplatelet therapies).
- Laboratory tests: CBC, renal function (BUN, creatinine, eGFR), fasting glucose & insulin, 14-day CGM, HbA1c, HOMA-IR & HOMA-β, lipid profile, liver function (ALT, AST, ALP, TBil, GGT), plasma catecholamines.
- HRV: 24-hour Holter monitoring.
- AE assessment and management (AE, SAE, SADE, UADE, USADE, ADE).

### 5.4.12 6-Month Follow-up (±30 days)

At 6 months post-procedure, subjects must return for follow-up including:

- Physical exam (height, weight, waist & hip circumference, office BP).
- Antidiabetic medication/insulin use.
- Other treatments (cardiovascular, anticoagulant, antiplatelet therapies).
- Laboratory tests: CBC, renal function (BUN, creatinine, eGFR), fasting glucose & insulin, 14-day CGM, HbA1c, HOMA-IR & HOMA-β, lipid profile, liver function (ALT, AST, ALP, TBil, GGT), plasma catecholamines.
- HRV: 24-hour Holter monitoring.
- AE assessment and management (AE, SAE, SADE, UADE, USADE, ADE).
- OGTT (5-point)
- 14-day CGM.
- Insulin and C-peptide secretion tests (5-point), HOMA-IR, HOMA-β.
- CTA and/or DSA to assess vascular patency.
- EQ-5D-5L quality of life questionnaire.

Note: Subjects with CTA showing >50% stenosis at EDN sites must undergo DSA. If CTA is normal, DSA is not required. Metformin must be withheld on the day of CTA or DSA.

### 5.4.13 12-Month Follow-up (±30 days)

At 12 months post-procedure, subjects must return for follow-up including:

- Physical exam (height, weight, waist & hip circumference, office BP).
- Antidiabetic medication/insulin use.
- Other treatments (cardiovascular, anticoagulant, antiplatelet therapies).
- Laboratory tests: CBC, renal function (BUN, creatinine, eGFR), fasting glucose & insulin, 14-day CGM, HbA1c, HOMA-IR & HOMA-β, lipid profile, liver function (ALT, AST, ALP, TBil, GGT), plasma catecholamines.
- AE assessment and management (AE, SAE, SADE, UADE, USADE, ADE).

.

### 5.4.14 24-Month Follow-up (±30 days)

At 24 months post-procedure, subjects must return for follow-up including:

- Physical exam (height, weight, waist & hip circumference, office BP).
- Antidiabetic medication/insulin use.
- Other treatments (cardiovascular, anticoagulant, antiplatelet therapies).
- Laboratory tests: CBC, renal function (BUN, creatinine, eGFR), fasting glucose & insulin, 14-day CGM, HbA1c, HOMA-IR & HOMA-β, lipid profile, liver function (ALT, AST, ALP, TBil, GGT), plasma catecholamines.
- AE assessment and management (AE, SAE, SADE, UADE, USADE, ADE).

### 5.4.15 End of Study

The study will conclude after all enrolled subjects have completed 24 months of follow-up, or have withdrawn (due to death or loss to follow-up), or exceeded the final visit window (>30 days). Data collected will be used to evaluate all study endpoints. Any subsequent adverse events will be managed according to standard clinical care by the treating physician.

###

### 5.4.16 Source Documents

Copies of preserved original paper records and printouts of original electronic records shall be declared as true copies of the source documents and must be signed and dated by a member of the study site team.

## 5.5 Device Use Specifications

### 5.5.1 Surgical Technique

This study uses the endovascular denervation (EDN) system (generator and catheter) manufactured by Shanghai Brattea Medical Technology Co., Ltd. to perform EDN in the celiac trunk, common hepatic artery, and/or proper hepatic artery for the treatment of T2DM. The operating physician and associated staff must receive adequate training on use of the investigational devices prior to the procedure and follow the surgical workflow below.

### 5.5.2 Surgical Workflow

#### 5.5.2.1 Pre-procedure Preparation

① Prepare the EDN generator, EDN catheter, and procedural consumables in advance. Place the generator in a position convenient for observation and operation, with the display facing the operator.

② Position the subject supine on the operating table. Firmly attach the neutral (return) electrode to the buttock. Perform routine disinfection and draping of the right groin.

#### 5.5.2.2 Pre**-**EDN Angiography

① Identify a puncture site ~1 cm inferior to the midpoint of the right groin; infiltrate with 2% lidocaine for local anesthesia.

② Perform right femoral arterial access using the Seldinger technique and place an 8F vascular sheath.

③ Advance a 5F pigtail catheter over a 0.035-inch hydrophilic (loach-type) guidewire through the sheath; position the catheter tip at the superior margin of T12 and perform abdominal aortography (DSA) to delineate the morphology and course of the celiac axis and branches, and to confirm whether angiographic exclusion criteria are present.

④ If no angiographic exclusion criteria are met, the subject may be enrolled in this clinical study.

⑤ Remove the pigtail catheter. Using a coaxial technique, advance a 5F hepatic artery catheter (Rh catheter) and an 8F guiding catheter over a 0.035-inch guidewire. Position the tip of the guiding catheter in the proper hepatic artery.

#### 5.5.2.3 EDN Procedure

Target vessels for ablation in this study are the celiac trunk, common hepatic artery, and/or proper hepatic artery. Steps:

① Determine target vessel anatomy and diameter by angiography to select an appropriately sized ablation catheter.

② After removing the Rh catheter, advance the 6-electrode EDN ablation catheter (manufactured by Shanghai Brattea Medical Technology Co., Ltd.) through the guiding catheter into the target vessel (see the product IFU for catheter operation).

③ Connect the RF system per the generator IFU and perform generator self-check.

④ Expand the distal basket and appose the electrodes to the vessel wall using the handle controls. Assess wall contact via displayed temperature and impedance (temperature ~37 °C, impedance <400 Ω) and adjust as needed.

⑤ Once adequate wall contact is confirmed, set RF parameters (temperature 60 °C; ablation time 120 s).

⑥ Begin RF ablation under IV analgesia/sedation (see Section 5.4.7.6). Continuously monitor real-time power, impedance, and temperature.

⑦ After each ablation, perform DSA to evaluate vessel morphology and detect complications. If vasospasm is observed, pause manipulation and reimage after 5 minutes. If spasm persists, slowly administer papaverine or nitroglycerin via the guiding catheter and reassess after 5 minutes.

⑧ Repeat RF ablation in the celiac trunk, common hepatic artery, and/or proper hepatic artery as required. Maintain adequate anticoagulation, sedation, and analgesia intraoperatively.

⑨ After completing ablation in all three segments, remove the ablation and guiding catheters; reintroduce the pigtail catheter and perform celiac angiography.

⑩ Upon confirming no significant abnormalities at ablation sites, remove all catheters. Close the arterial puncture using a Cordis vascular closure device/ProGlide suture-mediated device and apply local compression dressing. When dorsalis pedis or posterior tibial pulses are normal, the procedure is complete and the subject is transferred back to the ward.

# 6. Statistical Considerations

## 6.1 Primary Endpoints

Primary Safety Endpoint

Incidence of composite major adverse events (MAE)* related to the investigational device and/or ablation procedure occurring intraoperatively or within 30 days after the baseline procedure.

* MAE includes:

- Hypoglycemia or hyperglycemia requiring hospitalization.
- Vascular complications requiring surgical repair, interventional therapy, thrombin injection, or blood transfusion.
- Celiac/hepatic artery dissection or perforation requiring intervention.
- Significant embolic events causing end-organ damage or requiring intervention to prevent end-organ damage.
- Acute liver injury or liver failure.
- All-cause mortality.

**Primary Efficacy Endpoint**

Change in HbA1c at 6 months post-procedure compared with baseline.

## 6.2 Study Hypothesis

This is a single-arm feasibility clinical trial designed to explore the safety and efficacy of the EDN generator and catheter manufactured by Shanghai Brattea Medical Technology Co., Ltd. for the treatment of T2DM; therefore, no formal statistical hypothesis is specified.

## 6.3 Sample Size

A total of 30 subjects will be enrolled to support the study objectives.

## 6.4 Statistical Methods

**Statistical Analysis Sets**

Primary, secondary, and additional endpoints will be analyzed using both the Intent-to-Treat (ITT) and Per-Protocol (PPS) populations.

ITT: All subjects who signed written ICF and were enrolled, regardless of whether EDN was successfully performed.

PPS: Subjects who underwent EDN in the celiac/hepatic arteries using the investigational device.

**Control of Bias/Systematic Error**

Data from all study centers will be pooled for analysis.

**Enrollment by Center**

Given the small sample size of this feasibility trial, no upper limit is set per site; competitive enrollment will be used.

**Data Analysis**

As a single-arm feasibility study, no formal hypothesis testing is planned. Descriptive statistics will summarize baseline demographics, safety endpoints, and other categorical data for all enrolled subjects. For efficacy endpoints, paired t-tests will be used to compare post- vs. pre-procedure HbA1c (%) and other continuous outcomes, and the one-sided 97.5% lower confidence limit of the mean difference will be calculated.

**Interim Analysis**

No interim analysis is planned to stop early for efficacy or futility.

**Subgroup Analysis**

No prespecified subgroup analyses are planned due to the small sample size.

# 7. Monitoring Plan

The monitoring plan includes:

- Pre-study monitoring: investigational product, study documents, study personnel, and training.
- On-study monitoring: routine visits, submissions to the ethics committee during the trial, and AE monitoring.
- End-of-study (or early termination) monitoring: retrieval/destruction of investigational products, EDC/eCRF reconciliation and data queries, verification of document completeness, ethics notifications, site close-out, and clinical study report.

Monitors will conduct periodic on-site reviews to ensure full compliance with the approved protocol/amendments and will review source documents to verify eCRF/EDC entries.

The investigator/institution shall ensure that Shanghai Brattea Medical Technology Co., Ltd., its designees, and relevant regulatory authorities have direct access to source documents (electronic or paper). If original medical records from non-study institutions/providers are not accessible, certified copies of source documents must be provided for review.

# 8. Data Management

Data management will be performed by a CRO and will comply with applicable regulations and the DM department’s SOPs to ensure data are authentic, accurate, complete, and reliable.

## 8.1 Data Entry

Investigators shall collect subject data per protocol and complete the eCRF accurately, timely, completely, and规范ly per the data entry guidelines. The eCRF does not constitute a source document.

## 8.2 Database Setup and Data Verification

An Electronic Data Capture (EDC) system will be used and must comply with FDA 21 CFR Part 11, FDA software validation requirements, and relevant CFDA/NMPA guidance on EDC and clinical data management. eCRFs will be designed by Data Management based on the protocol.

A Data Verification Plan will be prepared according to requirements for completeness, consistency, and accuracy. Logical checks will be programmed/tested and performed via system-automated and manual reviews. Queries generated must be verified and resolved by the investigator or authorized staff.

Queries may also arise during source data review, medical coding, and SAE reconciliation; these processes continue until data cleaning is complete. After cleaning, investigators will electronically sign each eCRF.

The system must maintain an audit trail of any post-save data modifications (who, original value, timestamp, reason). Complete query/audit records shall be retained.

## 8.3 Database Lock

Following the lock procedure, once all pre-lock checks and QC are complete, database lock will be approved in writing; editing privileges and accounts will be revoked, and the Data Manager will lock the database. Any changes after lock must follow formal unlock/re-lock procedures.

After lock, a PDF of each subject’s eCRF containing study data and the audit trail will be generated and archived (e.g., on optical media). All database data and DM documentation will be archived appropriately. As a rule, no changes shall be made to the locked database/files.

### 8.4 Data Processing

After database lock, the statistical team will conduct the statistical analyses.

# 9. Risk–Benefit Analysis

## 9.1 Risks and Adverse Events Related to Endovascular Procedures

Risks are similar to those for patients undergoing comparable vascular interventions outside the study and may include, but are not limited to:

- Allergic reactions (to contrast agents, devices, medications, etc.)
- Access-site complications (bleeding, hematoma, AV fistula, pseudoaneurysm, infection, pain)
- Thrombosis/thrombus formation
- Embolization (air, tissue, thrombus, or material-related)
- Renal impairment or failure
- Pyrexia
- Infection/sepsis
- Vasospasm or occlusion
- Vascular injury (dissection, perforation, rupture, hemorrhage)
- Retroperitoneal hemorrhage
- Vascular complications requiring surgery
- Transient hemodynamic instability (hypotension/hypertensive episodes)
- Arrhythmias
- Cardiopulmonary arrest
- Death

## 9.2 Potential Risks Related to the Investigational Device and EDN

- Potential device/procedure-related risks include:
- Pain
- Celiac/hepatic arterial thrombosis or embolization and resultant end-organ injury
- Celiac/hepatic arterial dissection, perforation, rupture, or bleeding
- Vascular injury or aneurysm formation of the celiac/hepatic arteries
- Vasospasm or acute closure of the celiac/hepatic arteries
- Post-EDN stenosis at ablation sites in the celiac/hepatic arteries
- Hepatic dysfunction
- Gastrointestinal adverse reactions (nausea, vomiting, bloating, diarrhea, etc.)
- Orthostatic hypotension
- Post-procedure hypoglycemia
- Skin burns
- Other unforeseeable risks may also exist.

## 9.3 Study-Related Risks

In addition to the above, post-EDN antiplatelet therapy may increase bleeding risk. Additional unanticipated procedure-related risks may occur.

## 9.4 Risk Minimization Measures

Potential risks may exist. Adherence to the protocol, conducting procedures at qualified centers, comprehensive training of personnel on the device and procedure, strict screening, and close intra-/post-procedural monitoring with timely reporting of all required information to Shanghai Brattea Medical Technology Co., Ltd. will minimize risks.

## 9.5 Expected Benefits

Potential benefits include effective glycemic reduction for T2DM subjects whose glucose remains uncontrolled despite ≥1–3 OADs and/or insulin, with anticipated reductions in blood glucose/HbA1c after EDN.

## 9.6 Risk–Benefit Ratio

The EDN generator and catheter manufactured by Shanghai Brattea Medical Technology Co., Ltd. are expected to achieve the intended therapeutic effects. Based on current risk assessment, no unacceptable or intolerable residual risks have been identified; all applicable risks are described in the product IFU. Under the intended conditions of use, the anticipated benefits outweigh the risks.

# 10. Quality Control of the Clinical Trial

**Device Use Quality Control:**

Before trial initiation, the sponsor will provide technical personnel to train site staff on the proper operation of the investigational devices to ensure correct use.

**Investigator Training:**

Prior to trial initiation, the site principal investigator shall organize protocol training for all study investigators.

**Measures to Improve Subject Compliance:**

Investigators shall conduct the informed consent process diligently so that subjects fully understand the trial requirements and cooperate with study procedures.

**Clinical Trial Monitoring:**

Sponsor-appointed monitors will conduct regular on-site monitoring visits at each study center to ensure strict adherence to the protocol and to verify that entries in the eCRF are consistent with the source documents.

**Data Entry:**

Data entry and management are the responsibility of the Data Management Department. Data managers will program the data entry system and perform data entry and management. To ensure accuracy, double data entry will be performed independently by two data entry personnel, followed by reconciliation.

**Management of Devices/Equipment**

Investigational devices/equipment must be securely stored and used exclusively for this clinical trial. Devices shall be appropriately labeled as “For Investigational Use”.

The sponsor (Shanghai Brattea Medical Technology Co., Ltd.) will maintain records of device accountability, documenting the chain of custody from shipment to the study center through return or destruction.

The principal investigator or designee shall maintain records of receipt, use, return, and destruction, including:

- Date of receipt
- Batch/lot number or unique code identifying each device used in the trial
- Expiry date
- Date(s) of use
- Subject ID/code
- Date of return of used devices
- Date and quantity of unused, expired, or malfunctioning devices returned

# 11. Ethical Considerations and Informed Consent

## 11.1 Ethical Considerations

The clinical trial must be conducted in accordance with the Declaration of Helsinki and relevant national regulations and guidelines governing clinical research in China.

Before enrollment, investigators are responsible for providing each subject (or their legally authorized representative) with a written explanation of the study’s purpose, procedures, and potential risks. Subjects must be informed that they have the right to withdraw at any time. A written Informed Consent Form (ICF) must be provided and signed before any trial-related procedures are performed; the signed ICF must be retained in the trial files.

Prior to trial initiation at a site, the Ethics Committee (EC) shall review the ethical acceptability and scientific validity of the protocol, as well as feasibility at the institution (including investigator qualifications/experience and site equipment/capabilities).

The EC shall conduct ongoing oversight of the clinical trial at the institution. If subject rights and interests cannot be adequately protected, the EC may require the trial to be suspended or terminated at any time in writing. A suspended trial shall not resume without EC approval.

## 11.2 Protocol Approval

Before the trial begins, the investigator shall submit the clinical trial protocol, ICF, and related documents through the hospital’s clinical trial administration to the site’s EC for approval. The clinical trial may commence only after EC approval. Any protocol amendments must be approved by the EC prior to implementation.

## 11.3 Informed Consent Process and ICF Document

Participation is voluntary. When a subject is unable to decide for themselves (e.g., severe illness/coma, psychiatric disorders, intellectual disability), informed consent must be obtained from the subject or their legally authorized representative, and the subject shall be informed of the research to the extent compatible with their capacity. If the subject or legally authorized representative is illiterate, a neutral witness must participate in the process and sign the ICF. Investigators must ensure that informed consent is obtained before use of any investigational device, any study procedure, assessment, or data collection.

The process for obtaining and documenting informed consent must comply with ICH-GCP, the Declaration of Helsinki, ISO 14155, China NMPA regulations, and local EC/regulatory requirements. The ICF must receive EC approval.

**Key requirements for obtaining informed consent include:**

- Conducted by the principal investigator or an authorized designee;
- Providing a description of all relevant aspects of the clinical study;
- Avoiding coercion or undue influence on the decision to participate;
- Not waiving any of the subject’s legal rights;
- Communicating in language understandable to the subject or their legally authorized representative;
- Allowing adequate time for consideration and questions;
- Ensuring timely provision/updates of important information relevant to the subject throughout the study.

Informed consent must be signed by the subject or a legally authorized representative in accordance with applicable laws/regulations/guidelines, and under the responsibility of the investigator or authorized designee. If consent is initially provided by a legally authorized representative, the investigator shall seek consent from the subject as soon as feasible once the subject is capable. The site shall retain the original signed ICF; the subject shall receive a signed copy.

If new information emerges that may significantly affect a subject’s future health or medical care, the ICF must be revised promptly and the new information communicated to subjects. In some circumstances, enrolled subjects may need to re-consent to the revised ICF. In addition to important new information arising during the trial, revisions may also be required for major protocol amendments, change of principal investigator, administrative updates, or EC periodic review. All ICF revisions must receive EC approval. If the site EC requires changes to the revised ICF, approval from Shanghai Brattea Medical Technology Co., Ltd. is also required. The EC will determine whether re-consent is required for subjects already enrolled.

Investigators must explain that even after signing the ICF, screening or test results may ultimately determine that a subject is ineligible for the study. A screening log will be maintained to record screen failures, including (but not limited to) reasons for screen failure.

Any deviations related to the informed consent process must be reported to Shanghai Brattea Medical Technology Co., Ltd. and to local authorities (e.g., the EC) as required.

# 12. Requirements for Reporting Adverse Events and Device Deficiencies

## 12.1 Definitions of Adverse Events

This clinical trial adopts definitions based on (but not limited to) ISO 14155, EU MDR 2017/745 / MDCG 2020-10/1, U.S. FDA 21 CFR Part 812, and China NMPA GCP for Medical Device Clinical Trials (see Table 12-1).

**Table 12-1. Definitions of Adverse Events**

| **Category** | **Definition** |
| --- | --- |
| **Adverse Event (AE)** | Any untoward medical occurrence, unintended disease or injury, or any untoward clinical sign (including abnormal laboratory findings) in a subject, user, or other person in a clinical investigation, whether or not related to the investigational device and whether or not anticipated. Note 1: Includes AEs related to the investigational or comparator device. Note 2: Includes AEs related to associated procedures (any procedure required by this study). Note 3: For users or other persons, this definition applies only to events related to the investigational device. |
| **Adverse Device Effect (ADE)** | Any AE related to the use of the investigational medical device. Note 1: Includes AEs resulting from inadequate or inappropriate instructions, deployment/implantation/installation/operation, or device malfunction. Note 2: Includes events due to device use error or abnormal use. Remark 3: If a comparator is a medical device, AEs related to the comparator are also included. |
| **Serious Adverse Event (SAE)** | An AE that results in any of the following: • Death; • Serious deterioration in the health of the subject, user, or other person resulting in any of: – Life-threatening illness or injury; – Permanent impairment of a body structure or a body function, including chronic disease; – Inpatient hospitalization or prolongation of existing hospitalization; – Medical or surgical intervention to prevent life-threatening illness/injury or permanent impairment; • Fetal distress, fetal death, or a congenital anomaly/birth defect (including physical or intellectual defects). Note: Planned hospitalization for pre-existing conditions or protocol-required procedures without deterioration of health is not considered an SAE. |
| **Serious Adverse Device Effect (SADE)** | An ADE that has resulted in any of the consequences characteristic of an SAE. |
| **Unanticipated Adverse Device Effect (UADE)** | Any serious adverse effect on health or safety, life-threatening problem, or death caused by or associated with the device that was not previously identified in nature, severity, or incidence in the protocol or application (including supplements), or any other unanticipated serious problem associated with the device that relates to the rights, safety, or welfare of subjects. |
| **Unanticipated Serious Adverse Device Effect (USADE)** | A SADE whose nature, incidence, severity, or outcome is not identified in the current risk assessment. Note: An anticipated SADE (ASADE) is one whose nature, incidence, severity, or outcome is identified in the risk analysis report. |
| **Serious Public Health Threat** | Any signal of an AE or device deficiency that poses an imminent risk of death or serious deterioration in the state of health to subjects, users, or other persons, and requires prompt remedial action. Note: Includes serious, unexpected events raising concern for potential public health hazard or short-term risk of multiple deaths. |
| **Device Deficiency** | Inadequacy of a medical device with respect to its identity, quality, durability, reliability, usability, safety, or performance. Note 1: May include device malfunction, use error, or inadequate manufacturer information (including labeling). Note 2: Includes deficiencies of the investigational (or comparator) device. |

Clarification for “hospitalization” and “prolonged hospitalization” in SAE definition:

(1) Hospitalization does not include:

- Emergency treatment not resulting in admission.
- Note: Although emergency treatment itself is not “hospitalization,” it may still qualify as an SAE (e.g., intervention to prevent permanent harm).
- Elective/planned treatment or surgery for pre-existing conditions documented at consent/enrollment.
- Admissions for social reasons and/or temporary custodial care without health deterioration (e.g., homelessness).

• Protocol-required admissions (e.g., a planned study procedure).

Note: Complications or AEs occurring during elective/planned admissions after ICF signing must be reported as AE/SAE if they meet definitions; the original planned admission itself is not an SAE.

(2) Prolonged hospitalization means extension of the current admission beyond the expected standard length of stay for the treated condition.

Note: For new AEs during admission, assess whether they prolonged the stay or meet other SAE criteria.

## 12.2 Reporting of Adverse Events

Investigators shall assess and report to Shanghai Brattea Medical Technology Co., Ltd. the following:

- All AEs/SAEs;
- All device deficiencies/malfunctions;
- UADE/USADE;
- ADE/SADE;
- Any new findings/updates related to previously reported events.

Special attention shall be paid to MAE, 3MACE, and hypoglycemia/hyperglycemia, which must be promptly reported to the sponsor.

From the time of ICF signing and enrollment, any protocol-reportable AE occurring intra- or post-procedure must be documented in the eCRF and reported by medical diagnosis, not merely by symptoms. If the nature of an event is uncertain, or if it cannot be clearly separated from device/procedure factors, report it as an AE and/or device deficiency.

Notes: Pre-existing conditions are not reported as AEs unless severity or frequency increases during the study. Death is not reported as an AE per se; it is the outcome of a specific SAE and must be reported as such.

## 12.3 Causality Assessment with Investigational Device/Procedure

Investigators shall assess relatedness of each reported event to the investigational device and to the study procedure (per MDCG 2020-10/1, Table 12-2), and assess relatedness to antiplatelet therapy (Table 12-3).

**Table 12-2. Criteria for Assessing Relationship Between Investigational Device/Procedure and AE**

| **Relatedness** | **Definition** |
| --- | --- |
| **Not related** | The event can be excluded as related when one or more of the following apply: no temporal relationship with device use or procedure; reaction pattern inconsistent with known device effects and biologically implausible; stopping/decreasing or re-using/increasing exposure has no effect (when clinically feasible); involves a body part not affected by device/procedure; explained by other causes (baseline disease/comorbidity, another device/medication/therapy, other risk factors); if applicable, unrelated to an erroneous diagnostic result from the device. (Not all conditions are required; depends on device/procedure and event type.) |
| **Possibly related** | Weak association to device/comparator use or procedure cannot be excluded; may be attributable to other factors (baseline disease/comorbidity and/or another device/medication/therapy). When relatedness cannot be assessed or information is pending, classify as possibly related. |
| **Probably related** | Event appears related to device use or procedure and/or cannot be reasonably explained by another cause. |
| **Causally related** | Relationship is beyond reasonable doubt when (as applicable): known side effect of this product class or similar devices/procedures; temporal relationship present; involves body part affected by or influenced by the device/procedure; follows known reaction pattern; de-challenge/re-challenge affects the event (when feasible); alternative causes adequately excluded; injury due to use error; if applicable, caused by erroneous diagnostic result from the device. (Not all items are required; depends on device/procedure and event type.) |

**Table 12-3. Criteria for Assessing Relationship Between Antiplatelet Therapy and AE**

| **Relatedness** | **Definition** |
| --- | --- |
| **Not related** | Exclude relatedness when: no temporal relationship with antiplatelet use; reaction pattern inconsistent with known effects and biologically implausible; de-challenge/re-challenge has no effect (when feasible); involves body part not affected by antiplatelets; explained by other causes (baseline disease/comorbidity and/or another device/medication/therapy). (Not all conditions are required; depends on drug and event type.) |
| **Possibly related** | Weak association to antiplatelet use cannot be excluded; other factors may contribute. If assessment is not possible or information is pending, classify as possibly related. |
| **Probably related** | Event appears related to antiplatelet use and/or not reasonably explained by another cause; further information may be needed. |
| **Causally related** | Relationship is beyond reasonable doubt when: known side effect of the pharmacologic class; temporal relationship present; involves an affected body part; follows known reaction pattern; de-challenge/re-challenge affects the event (when feasible); alternative causes excluded; injury due to medication use error. (Not all items are required; depends on drug and event type.) |

## 12.4 Investigator Reporting Requirements

Investigators shall report events and device deficiencies to the sponsor per Table 12-4.

**Table 12-4. Investigator Reporting Requirements**

| **Category** | **Reporting Method** | **Reporting Timeline** |
| --- | --- | --- |
| **UADE / USADE** | Complete the eCRF AE form using all available new/updated information. | Within 24 hours of first awareness, or per local/regional regulations; continue reporting until end of study. |
|  | Provide all source documents related to the event (with identifiers removed). | As requested by the sponsor. |
| **SAE (including SADE)** | Complete the eCRF AE form using all available new/updated information. | Within 24 hours of first awareness, or per local/regional regulations; continue reporting until end of study. |
|  | For SADE, provide all related source documents (de-identified). | When source docs are available. |
| **Device deficiency (including but not limited to failure, malfunction, use error, nonconformity, labeling error). Note: Even without an AE, any device deficiency that could have led to an SAE if a) appropriate action had not been taken; or b) intervention had not occurred; or c) circumstances had been less fortunate, is reportable.** | Complete the eCRF Device Deficiency form using all available information. | Within 24 hours of first awareness, or per local/regional regulations; continue reporting until end of study. |
| **AE (including ADE)** | Complete the eCRF AE form, including AE onset date, treatment, severity, and assessment of relatedness to device. | Report promptly upon awareness, no later than 10 working days; continue reporting until end of study. |

Abbreviations: AE = adverse event; eCRF = electronic case report form; ADE = adverse device effect; SAE = serious adverse event; SADE = serious adverse device effect.

## 12.5 Device Deficiencies

All investigational device deficiencies (including failure, malfunction, use error, nonconformity, and labeling error) must be documented and reported to Shanghai Brattea Medical Technology Co., Ltd. When feasible, the device should be returned to the sponsor for analysis. If return is not possible, record the reason and the device’s final disposition. Device failures and inability to function should also be documented in the subject’s medical record.

A device deficiency alone is not reported as an AE. However, if a device deficiency results in an AE, the corresponding AE must be recorded in the eCRF. In addition, any device deficiency that could have led to an SAE under less favorable circumstances must be reported per Table 12-4.

## 12.6 Sponsor Reporting Requirements

Shanghai Brattea Medical Technology Co., Ltd. is responsible for reporting AE and device deficiency information to all participating investigators, ethics committees, and regulatory authorities (per Chinese domestic requirements). Principal Investigators must report UADE/USADE and SAE to the EC and regulators per local regulations.

For serious adverse events and device deficiencies that could lead to an SAE, the sponsor shall, per domestic requirements:

Report within 7 days after becoming aware of an investigational device-related death or life-threatening SAE;

Report within 15 days after becoming aware of non-fatal, non-life-threatening investigational device-related SAEs and other serious safety risks;

to: other participating sites, ECs, principal investigators, the drug/device regulatory authority at the sponsor’s location (province/autonomous region/municipality), and the authorities at the clinical site’s location (drug regulatory and health authorities).

If any SAE/SADE or device deficiency suggests increased procedural/device risk, or protocol/process changes are needed to mitigate unanticipated risks, the sponsor shall notify all participating sites.

Per domestic requirements, the sponsor, investigators, or sites must report all UADE, USADE, SADE, SAE, and device deficiencies to the EC. Reports submitted to the EC (and other applicable reports) must also be provided to the sponsor.

# 13. Protocol Deviations

Except in emergency situations where it is necessary to protect the life and health of the subject, investigators shall not change or deviate from this clinical trial protocol. Investigators must promptly notify the sponsor (Shanghai Meiliweiye Medical Technology Co., Ltd.) and the ethics committee of any protocol deviations made in emergency situations to protect the life or health of subjects. Such notifications should be issued as soon as possible after the emergency, generally no later than five working days, or earlier as required by local regulations.

All protocol deviations, including the cause and date of occurrence, must be recorded in the EDC system and reported to the sponsor. According to national guidelines and government regulations, the research center must also report protocol deviations to the ethics committee.

The sponsor will continuously review and evaluate protocol deviations and, if necessary, take appropriate corrective and preventive measures (including, but not limited to, notifications, retraining of the study center, or termination of the study center’s qualification).

**Classification of protocol deviations:**

**Major protocol deviation** refers to deviations that directly or potentially affect the conduct of the study (i.e., impact study design, data, and results), or deviations that affect subject safety and rights.

**Minor protocol deviation** refers to deviations that do not jeopardize the progress of the study (i.e., study design, data, and results remain unaffected) and do not endanger the safety and rights of subjects.

The sponsor shall ensure that all investigators conducting the clinical trial strictly adhere to the protocol. If clinical trial institutions or investigators fail to comply with relevant laws and regulations, China’s Medical Device GCP, or the protocol, the sponsor shall promptly point out and correct such issues; if the situation is serious or remains uncorrected, the trial shall be terminated, and the sponsor shall report to the local provincial/municipal food and drug administration as well as the National Medical Products Administration (NMPA).

# 14. Direct Access to Source Data and Documents

In this clinical trial, the principal investigator and authorized personnel may have direct access to source data and documents.

Access to and editing rights of source data by other personnel shall comply with the relevant Chinese laws, regulations, technical standards, and the detailed requirements outlined in the applicable SOPs of relevant departments.

# 15. Contents to be Included in the Clinical Trial Report

According to the requirements of the Good Clinical Practice for Medical Device Clinical Trials (2022) jointly issued by the National Medical Products Administration (NMPA) and the National Health and Family Planning Commission, the clinical trial report should include the following contents:

- General information
- Abstract
- Introduction
- Objectives of the clinical trial
- Clinical trial methodology
- Clinical trial content
- General clinical data
- Diagnostic and therapeutic methods of investigational and control products
- Statistical analysis and evaluation methods used
- Clinical evaluation criteria
- Organizational structure of the clinical trial
- Statement of ethical considerations
- Clinical trial results
- Adverse events observed during the trial and their management
- Analysis and discussion of clinical trial results, particularly indications, scope of application, contraindications, and precautions
- Conclusions of the clinical trial
- Existing problems and suggestions for improvement
- List of trial personnel
- Other issues requiring explanation

# 16. Confidentiality Principles

This clinical trial protocol is confidential information, provided only to medical experts involved in the trial, participating investigators, relevant staff, medical institutions undertaking the trial, ethics committees, and contract research organizations or related service providers. Except when informing subjects, no part of this protocol may be disclosed to or shared with any third party without the prior written consent of the sponsor. Furthermore, partial or full trial results intended for presentation at conferences, journals, or other external outlets must obtain prior written approval from the sponsor.

This agreement, the clinical trial content, and all associated materials are confidential and exclusively owned by the sponsor. Investigators bear the responsibility of maintaining confidentiality. This includes patent applications, manufacturing processes, and unpublished data provided by the sponsor for research use. Such information must not be disclosed to any third party without sponsor approval. This confidentiality obligation remains effective even after the termination or completion of the trial.

Investigators and their staff will collect subjects’ personal data for the study (“study data”), including date of birth, sex, identification number, home address, and personal information regarding physical or mental health status.

All medical records and study materials that can identify subjects will be kept confidential to the extent permitted by law. However, the investigator, sponsor and representatives, monitors, and—under specific circumstances—regulatory authorities and ethics committees may inspect and copy confidential information containing identifiable subject data. All personal information in this study will be processed in accordance with national and local data protection laws.

Subjects have the right to request that investigators and the sponsor protect their information and correct any inaccuracies. If a subject withdraws informed consent, the investigator will no longer use or disclose the subject’s data. However, the sponsor may continue to use study data collected prior to withdrawal of consent.

The results of this study may be published in medical journals and presented at scientific meetings. In all such publications, subject identity will remain confidential.

# 17. Responsibilities of the Parties

## 17.1 Responsibilities of the Investigator

The Principal Investigator (PI) at each study center is responsible for ensuring that the study is conducted in accordance with the Clinical Investigation Agreement, the Protocol, ISO 14155 (or ICH/GCP), ethical principles based on the Declaration of Helsinki, the conditions of approval required by the Ethics Committee (EC), and applicable Chinese regulations (with the most protective rule for subjects prevailing).

**The PI’s responsibilities include, but are not limited to:**

- Signing the Clinical Investigation Agreement and the protocol signature page before study start, confirming the PI’s agreement to conduct the study per protocol.
- Providing evidence of qualifications and experience sufficient to reasonably undertake the study, as well as up-to-date CVs of key team members and disclosure of any potential conflicts of interest (including financial) that might interfere with conduct or results.
- Not changing or deviating from the protocol except in emergencies necessary to protect life or physical health of subjects. Documenting and explaining any departures and/or deviations from the approved protocol during the investigation.
- Creating and maintaining source documents for the entire study, ensuring monitors/auditors have direct access, and retaining all study-related records as required.
- Ensuring accuracy, completeness, clarity, and timeliness of CRF data and all other sponsor-required reports.
- Recording, reporting, and assessing (severity and relatedness to device/procedure) all AEs and observed device deficiencies.
- Reporting to Shanghai Brattea Medical Technology Co., Ltd. all AEs/SAEs, ADEs/SADEs, UADEs/USADEs, and all device deficiencies per protocol.
- Where required by national regulations, the protocol, or the EC, reporting all SAEs and device deficiencies that lead to SADE or may constitute UADE/USADE to the EC/regulators, and providing the sponsor with additional safety information as requested.
- Permitting sponsor monitoring and auditing; hosting monitors/auditors during visits and responding to queries.
- Allowing and supporting inspections/audits by regulatory authorities and the EC.
- Ensuring informed consent is obtained per the protocol and site EC requirements.
- Providing adequate medical care to subjects experiencing AEs during and after participation, as described in the protocol.
- Informing subjects of the nature and possible causes of AEs.
- Informing subjects of significant new findings during the study, including any additional treatments that may be required.
- Providing appropriate procedures for handling medical emergencies related to the study and arranging urgent care as needed.
- Ensuring medical records clearly indicate the subject’s participation in this study.
- When applicable, providing subjects with proof of participation and information on concomitant therapies/allowable treatments (with contact details).
- Notifying a subject’s primary physician of participation when approved by the subject or required by law.
- Respecting subjects’ rights and making reasonable efforts to ascertain reasons for any early withdrawal.
- Ensuring the site has sufficient personnel and equipment, and that study information and data can be recorded/retained during the investigation.
- Ensuring equipment used for study assessments is adequately maintained and calibrated, with records where applicable.

**Delegation of Duties**

When tasks (including the informed consent process) are delegated, the PI remains responsible for adequate training and supervision of designees. The PI is accountable for regulatory noncompliance resulting from insufficient oversight.

##

## 17.2 Ethics Committee (EC)

Before recruiting subjects, the sponsor must receive written EC and/or regulatory approval of the protocol and ICF. Recruitment materials and any other information provided to subjects must also be EC-approved.

Any protocol amendments must receive EC approval prior to implementation; all ICF changes require EC approval, and the EC shall determine whether re-consent with the revised ICF is required for subjects who signed a prior version.

Per Chinese regulations or EC requirements, annual continuing approval/re-approval must be obtained throughout the study. Copies of investigator reports and EC re-approvals must be provided to the sponsor.

## 17.3 Responsibilities of the Sponsor

Shanghai Brattea Medical Technology Co., Ltd. may utilize a CRO or other contractors as designees to perform specified tasks. Responsibilities of such entities shall be defined by appropriate contracts/agreements. The sponsor and its designees will treat all information and data obtained about subjects and their participation as confidential; only authorized sponsor personnel may access these records. Authorized regulatory personnel may inspect and copy all study-related records. Data collected during the study may be used by the sponsor for research purposes, publications, support of future research, and/or other legitimate business purposes. No subject will be identified by name in analyses or reports.

The sponsor will comply with all applicable regulations and device GCP, and will protect the confidentiality of health information. The sponsor may use health information for this study and for other purposes such as oversight and improvement of device performance, new medical research, development of new medical products/procedures, and other legitimate business purposes. Information received will not be used to market products to subjects, nor will subject identifiers be placed on mailing lists or sold for commercial use.

# 18. Insurance

In accordance with Chinese regulations, Shanghai Brattea Medical Technology Co., Ltd. will provide insurance for subjects in this study. In the event of study-related injury, the site will handle claims per the applicable insurance procedures; the sponsor will assume responsibility according to the policy terms, except for injuries caused by protocol violations or willful/mgross negligence of the site (see Clinical Trial Agreement).

# 19. Statement of Compliance

This study will be conducted in accordance with ISO 14155 for clinical investigations of medical devices in human subjects, ethical requirements based on the Declaration of Helsinki and GCP. The study may commence only after approvals/opinions have been obtained from the EC and/or regulatory authorities. All additional EC or regulatory requirements, where applicable, must be followed.

# 20. Committees

## 20.1 Executive Committee

An Executive Committee composed of the sponsor’s clinical management team, the Principal Investigator, and Co-Investigators will be convened. This committee will oversee overall conduct, including protocol design, study progress, subject safety, overall data quality and integrity, and timely dissemination of results through appropriate scientific meetings and publications. Additional study investigators may be invited as appropriate.

## 20.2 Safety Oversight Process

To enable early detection of safety issues, the sponsor and its authorized CRO safety team will review, process, monitor, and evaluate safety events defined in the study-specific Safety Plan. Timely, dynamic capture of unmonitored data following initial event reports is required. During routine monitoring visits, CRAs will support dynamic reporting by reviewing source documents and other data. The sponsor/CRO safety team will assess and classify events per the definitions of adverse events described above.

# 21. Study Suspension or Termination

## 21.1 Early Termination by the Sponsor

For scientific, administrative, or subject-protection reasons, the sponsor reserves the right to suspend or terminate the study at any time. If suspension/termination is required, the sponsor will notify investigators, relevant ECs, and regulatory authorities (as applicable) in writing, and provide instructions on the management of enrolled subjects.

**Criteria/reasons (not limited to):**

- Occurrence of UADE creating significant or unreasonable risks to enrolled subjects;
- Enrollment markedly below expectations, risking biased conclusions;
- Sponsor decision to suspend or discontinue development of the investigational device.

Note: Per Chapter 20 requirements, even if the study is terminated, evaluation/reporting of AEs, SAEs, SADEs, and device deficiencies for subjects who received the device must continue.

## 21.2 Termination by Investigator/Site or EC Withdrawal

Any participating investigator or site EC may terminate participation or withdraw approval, with immediate written notice to the sponsor. The PI or designee shall return all study documents and investigational devices to the sponsor.

In such cases, the sponsor will provide instructions on management of enrolled subjects and will notify other investigators, relevant ECs, and regulatory authorities (as applicable).

## 21.3 Criteria for Suspending/Terminating Site Participation

If a site remains without enrollments after enrollment has opened, or exhibits repeated or serious protocol violations without effective remediation, the sponsor may terminate the site’s enrollment privileges at any time after SIV and notify the EC and local regulators (as applicable). Subjects already enrolled at that site will continue the protocol-required follow-up.

# 22. Publication Policy

All data generated by this study are the property of the sponsor and are confidential. The sponsor reserves the right to publish study results and to allocate authorship per the publication policy described in the Clinical Trial Agreement.

# **23.** References

1. IDF Diabetes Atlas, 10th Edition Committee: IDF Diabetes Atlas 2021 – 10^TH^ Edition. [www.diabetesatlas.org](http://www.diabetesatlas.org)
2. 中华医学会糖尿病学分会. 中国2型糖尿病防治指南（2020年版）[J]. 中华糖尿病杂志, 2021,13(4): 315-409.
3. Zheng Y, Ley SH, Hu FB. Global aetiology and epidemiology of type 2 diabetes mellitus and its complications. Nat. Rev. Endocrinol. 2018, 14, 88–98.
4. World Health Organization 2016: Global report on diabetes - Global burden of diabetes.
5. Galicia-Garcia U, Benito-Vicente A, JebariInt S, et al. Pathophysiology of Type 2 Diabetes Mellitus. J. Mol.
6. Stratton IM, Adler AI, Neil HA, et al. Association of glycaemia with macrovascular and microvascular complications of type 2 diabetes (UKPDS 35): prospective observational study[J]. BMJ, 2000,321(7258): 405‑412.
7. Holman RR, Paul SK, Bethel MA, et al. 10‑year follow‑up of intensive glucose control in type 2 diabetes[J]. N Engl J Med, 2008, 359(15): 1577‑1589.
8. 中华医学会糖尿病学分会, 中华医学会内分泌学分会. 中国成人2 型糖尿病患者糖化血红蛋白控制目标及达标策略专家共识[J]. 中华糖尿病杂志, 2020, 12(1):1‑12.
9. Giugliano, D., Maiorino, M. I., Bellastella, G. & Esposito, K. Clinical inertia, reverse clinical inertia, and medication non-adherence in type 2 diabetes. J. Endocrinol. Investig. 2019;42:495–503.
10. Davies MJ, D'Alessio DA, Fradkin J, et al. Management of hyperglycaemia in type 2 diabetes, 2018. A consensus report by the American Diabetes Association (ADA) and the European Association for the Study of Diabetes (EASD). Diabetologia 2018; 61:2461–2498.
11. Carnagarin R, Kiuchi MG, Goh G, et al. Role of the sympathetic nervous systemin cardiometabolic control: implications for targeted multiorgan neuromodulation approaches. J Hypertens. 2021;39(8):1478-1489.
12. Thorp AA, Schlaich MP. Relevance of sympathetic nervous system activation in obesity and metabolic syndrome. J Diabetes Res 2015; 2015:341583.
13. Schlaich M, Straznicky N, Lambert E, Lambert G. Metabolic syndrome: a sympathetic disease? Lancet Diabetes Endocrinol 2015; 3:148–157.
14. Huggett RJ, Scott EM, Gilbey SG, Stoker JB, Mackintosh AF, Mary DA. Impact of type 2 diabetes mellitus on sympathetic neural mechanisms in hypertension. Circulation 2003; 108:3097–3101.
15. Landsberg L. Insulin-mediated sympathetic stimulation: role in the pathogenesis of obesity-related hypertension (or, how insulin affects blood pressure, and why). J Hypertens 2001; 19:523–528.
16. Symplicity HTN-1 Investigators. Catheter-based renal sympathetic denervation for resistant hypertension: durability of blood pressure reduction out to 24 months. Hypertension. 2011 May;57(5):911-7.
17. Symplicity HTN-2 Investigators. Renal sympathetic denervation in patients with treatment-resistant hypertension (The Symplicity HTN-2 Trial): a randomised controlled trial. Lancet.2010;376:1903-1909.
18. Mahfoud F, Schlaich M, Kindermann I, et al. Effect of renal sympathetic denervation on glucose metabolism in patients with resistant hypertension: a pilot study. Circulation 2011; 123:1940–1946.
19. Witkowski A, Prejbisz A, Florczak E, et al. Effects of renal sympathetic denervation on blood pressure, sleep apnea course, and glycemic control in patients with resistant hypertension and sleep apnea. Hypertension 2011; 58:559–565.
20. Schlaich MP, Straznicky N, Grima M, et al. Renal denervation: a potential new treatment modality for polycystic ovary syndrome? J Hypertens 2011; 29:991–996.
21. Verloop WL, Spiering W, Vink EE, et al. Denervation of the renal arteries in metabolic syndrome: the DREAMS-study. Hypertension 2015; 65:751–757.
22. Pan T, Guo JH, Ling L, et al. Effects of Multi-Electrode Renal Denervation on Insulin Sensitivity and Glucose Metabolism in a Canine Model of Type 2 Diabetes Mellitus. J Vasc Interv Radiol. 2018;29(5):731-738.e2. Doi: 10.1016/j.jvir.2017.12.011.
23. Zhang Q, Guo JH, Zhu HD, et al. Endovascular Denervation: A New Approach for Cancer Pain Relief? J Vasc Interv Radiol. 2018 Dec;29(12):1639-1644.
24. Pan T, Li L, Wei Q, et al. Endovascular Celiac Denervation for Glycemic Control in Patients with Type 2 Diabetes Mellitus. J Vasc Interv Radiol. 2021 Nov;32(11):1519-1528.e2. doi:10.1016/j.jvir.2021.07.023.
25. Kraft G, Vrba A, Scott M, et al. Sympathetic Denervation of the Common Hepatic Artery Lessens Glucose Intolerance in the Fat- and Fructose-Fed Dog. Diabetes 2019;68:1143–1155.
26. Kraft G, Scott M, Allen E, et al. Safety of surgical denervation of the common hepatic artery in insulin-resistant dogs. Physiological Reports. 2021;9:e14805.

**Investigator’s Declaration**

I hereby agree:

- To conduct this clinical investigation strictly in accordance with the Declaration of Helsinki, current Chinese regulations, and the requirements of the study protocol.
- To accurately record all required data in the Case Report Forms (CRFs) and complete the clinical study report on time.
- To use the investigational device samples solely for this clinical investigation, to maintain complete and accurate records of device receipt and use during the trial, and to retain such records.
- To permit monitoring, auditing, and inspection of this clinical investigation by monitors, auditors, and regulatory authorities authorized or appointed by the sponsor.
- To strictly comply with the terms of the clinical trial contract/agreement signed by all parties.

I have read the entire clinical study protocol, including the above declaration, and I fully agree to all of the above.

| Sponsor’s Statement  Signature (Seal):  　　　　　　　　　　　　　　 　Date: |
| --- |

| Investigator’s Statement  Signature (Seal):  　　　　　　　　　　　　　　 　Date: |
| --- |

| Statement of the Medical Device Clinical Trial Institution  Signature (Seal):  　　　　　　　　　　　　　　 　Date: |
| --- |
